# Microglial-specific depletion of TAK1 is neuroprotective in the acute phase after ischemic stroke

**DOI:** 10.1007/s00109-020-01916-9

**Published:** 2020-05-07

**Authors:** Thomas Zeyen, Rozina Noristani, Shahin Habib, Ole Heinisch, Alexander Slowik, Michael Huber, Jörg B. Schulz, Arno Reich, Pardes Habib

**Affiliations:** 1 Department of Neurology, RWTH Aachen University, Pauwelsstraße 30, D-52074 Aachen, Germany; 2 Medical Biochemistry, Department of Biochemistry, University of Leicester, Leicester, UK; 3 Institute of Neuroanatomy, Medical Faculty, RWTH Aachen University, Aachen, Germany; 4 Institute of Biochemistry and Molecular Immunology, Medical Faculty, RWTH Aachen University, Aachen, Germany; 5 JARA-BRAIN Institute of Molecular Neuroscience and Neuroimaging, Forschungszentrum Jülich GmbH and RWTH Aachen University, Aachen, Germany

**Keywords:** Ischemia-reperfusion injury, Stroke, Microglia, TAK1, Neuroinflammation

## Abstract

**Abstract:**

Transforming growth factor-β-activated kinase 1 (TAK1) is upregulated after cerebral ischemia and contributes to an aggravation of brain injury. TAK1 acts as a key regulator of NF-ΚB and the MAP kinases JNK and p38 and modulates post-ischemic neuroinflammation and apoptosis. Microglia are the main TAK1-expressing immunocompetent cells of the brain. However, little is known about the function and regulation of microglial TAK1 after cerebral ischemia. Tamoxifen-dependent conditional depletion of TAK1 in microglial cells was induced in *Cx3cr1*^*creER*^*-Tak1*^*fl/fl*^ mice. The *cre*^*ER*^-negative *Tak1*^*fl/fl*^ mice and vehicle-treated (corn oil) mice served as control groups. A transient intraluminal middle cerebral artery occlusion of 30 min followed by 6 h and 72 h of reperfusion was performed in male mice. Oxygen-glucose-deprivation (OGD) was performed with primary cortical glial cell cultures to examine the effect of microglial-specific and general (5Z-7-Oxozeaenol) TAK1 inhibition after different reperfusion times (1 h, 6 h, and 72 h). *Cx3cr1*^*creER*^*-Tak1*^*fl/fl*^ mice showed reduced infarct sizes and improved neurological outcomes compared to the control group. The mRNA and protein levels of pro-inflammatory *Il1b*/IL-1β and *Tnf*/TNF-α in the peri-infarct zones of microglial-specific TAK1-depleted mice were significantly reduced. Furthermore, TAK1 depletion in vitro led to reduced cell death rates after OGD. Moreover, hypoxia-mediated activation of TAK1 and its downstream signalling proteins, JNK and p38, were dampened by microglial TAK1 depletion. In contrast, 5Z-7-Oxozeaenol-induced pharmacological inhibition of TAK1 completely diminished MAPK-signalling including the kinases JNK and p38 in all cells. Microglial TAK1 depletion abrogates post-ischemic neuroinflammation and apoptosis in the acute phase, hence might be considered as a potential target in the treatment of cerebral hypoxia.

**Key messages:**

- TAK1 is activated after cerebral ischemia and induces MAP kinases p38 and JNK*.*
- Activated TAK1 increases apoptosis rate and the level pro-inflammatory cytokines IL-1β and TNF-α.
- Microglial cells seem to be the main source of TAK1-mediated post-ischemic neuroinflammation.
- Microglial-specific TAK1-depletion mediates sustainable neuroprotective effects, which might be superior to global TAK1 inhibition.

**Electronic supplementary material:**

The online version of this article (10.1007/s00109-020-01916-9) contains supplementary material, which is available to authorized users.

## Introduction

Ischemic stroke is one of the major causes of death and long-term disability in adults in most developed countries [1, 2]. Cerebral ischemia not only causes necrotic cell death in the infarct core, but also delayed apoptosis in the adjacent penumbra region. The latter is amenable to therapeutic strategies [3–5]. The post-ischemic injury initiates a cascade of inflammatory and pro-apoptotic events, which aggravate the damage and in turn determine the recovery after stroke [6, 7]. Understanding the regulation of post-ischemic neuroinflammation and cellular interplay holds much promise for potential clinical interventions [8, 9]. Following cerebral ischemia, NF-ΚB and the MAP kinases JNK and p38 are major regulators of pro-inflammatory gene transcription [10–13]. The mitogen-activated protein kinase kinase kinase (MAP3K) transforming growth factor-β-activated kinase 1 (TAK1; MAP3K7) is a 67-kDa protein acting as a common upstream activator of various kinases, such as JNK, p38, and IKK [14–16]. Ischemia-dependent TAK1 activation is known to increase expression of IL-1β, TNF-α, and IL-6 [17, 18]. An excessive production of the latter activates inflammatory and apoptotic signalling in the brain resulting in progressive tissue damage [19, 20].

Several studies describe diverse regulatory effects of TAK1 in different disease models indicating its “dual sword” function. TAK1 activation might be either harmful or pro-survival, depending on the stimulus and the cell type [14, 15, 21]. TAK1 acts in a pro-apoptotic manner in mouse embryonic fibroblasts (MEFs), but also enhances cell growth in neutrophils and increases inflammation and cell death in keratinocytes [17, 22]. General TAK1 inhibition using 5Z-7-Oxozeaenol has already been shown to exert neuroprotective effects in a model of subarachnoid hemorrhage and cerebral ischemia [23–25]. The application of 5Z-7-Oxozeaenol intracerebroventricularly or intraperitoneally in mice exerted dose-dependent and prolonged neuroprotective effects, including reduced infarct volumes and less neurological deficits after transient intraluminal middle cerebral artery occlusion (tMCAo) [25]. However, there is an evidence of an acute inhibition of TAK1 (intracerebroventricular injection of 5Z-7-Oxozeaenol 20 min prior to tMCAo), but not a chronic inhibition of TAK1 (5Z-7-Oxozeaenol treatment over 10 days) is neuroprotective in mice. The same authors demonstrated a lack of protective effects of neuronal-specific TAK1-depletion in mice after tMCAo [24].

Microglia, the resident immunocompetent cells of the central nervous system (CNS) play a pivotal role in the early phase of post-ischemic inflammation [26]. However, there is still disagreement regarding microglial function, whether they are neurotoxic or neuroprotective after cerebral ischemia [27, 28]. Their ability to phagocytose cell detritus and their essential regulatory function in inflammatory and apoptotic cell signalling is essential for recovery of damaged brain tissue [29]. Besides the production of growth factors such as brain-derived neurotrophic factor or eliminating dead tissue and debris after ischemia, microglial cells are also capable of releasing pro-inflammatory cytokines (TNF-α, IL-1 β, Il-6) and/or reactive oxygen species [8]. The role of TAK1 in microglia after cerebral ischemia has not been elucidated yet.

To address the function of TAK1 in microglial cells after cerebral ischemia, we induced microglial-specific TAK1 depletion in our conditional knockout mice after s.c. tamoxifen-injection [30]. We subjected *Cx3cr1*^*creER*^*-Tak1*^*fl/fl*^ mice and their cre^ER^-negative *Tak1*^*fl/fl*^ littermates to 30 min of tMCAo followed by 6 h or 72 h of reperfusion. Besides infarct sizes and neurological outcome, we also examined the post-ischemic neuroinflammation/apoptosis and the involved TAK1-dependent downstream signalling cascades. To investigate and to compare the time- and dose-dependent effect of general and cell-specific TAK1 depletion/inhibition, we utilized oxygen-glucose-deprivation (OGD) in mixed glial cortical cell cultures.

## Material and methods

All experimental procedures and protocols were approved by the Animal Care Committee of the RWTH Aachen University and by the District Government of North Rhine Westphalia in Recklinghausen, Germany (LANUV ID 84-20.04.2015.A292) and comply with the ARRIVE-guidelines.

### Animals

All mice were housed and handled in accordance with the guidelines of the Federation for European Laboratory Animal Science Associations (FELASA) in a pathogen-free, temperature-controlled (20–24 °C) facility with a 12/12-h light/dark cycle and access to pelleted food and water ad libitum. *Cx3cr1*^*CreER*^ mice (B6J.B6N(Cg)-*Cx3cr1*^*tm1.1(cre)Jung*^/J; Stock No: 025524, Jackson laboratory) were kindly provided by Tobias Goldmann and Marco Prinz (University of Freiburg, Germany) and maintained on a C57BL/6J background [30]. Mice carrying loxP-site–flanked (floxed) alleles (exon 2) of the TAK1-encoding gene *Map3k7* (*Tak1*^*fl/fl*^) were crossed to *Cx3cr1*^*CreER*^ mice (Jackson Laboratories). For induction of Cre recombinase, 5- to 7-week-old mice were treated with 4 mg tamoxifen (TAM, Sigma) dissolved in 200 μl corn oil (Sigma) injected subcutaneously two times in a period of 48 h. In all experiments, littermates carrying the respective loxP-flanked alleles but lacking expression of Cre recombinase (“*Tak1*^*fl/fl*^”) and vehicle-treated mice served as controls, respectively. For a better understanding, we provide an illustration of the conditional knockout model in supplemental Fig. 1A. Mice were bred by the Institute of laboratory animal science, Medical Faculty, RWTH Aachen University.

### Study protocol and animal surgery

A total of 74 C57BL/6J mice (10 to 12 weeks of age, weight 25–30 g) were randomly supplied to the surgeon for this randomized controlled trial (RCT, supplemental Fig. 1C). To prevent the potential effects of gonadal steroid hormones on post-ischemic inflammation as previously described [31, 32], we only subjected male mice to tMCAo or sham surgery*.* A technical assistant who was not involved in the analysis identified the mice by earmark numbers and randomly (QuickCalcs, Graphpad prism 6.0) assigned the mice to the experimental groups. Intraluminal transient middle cerebral artery occlusion (tMCAO) or sham surgery was performed for 30 min in 30 *Cx3cr1*^*creER*^-*Tak1*^*fl/fl*^ and 30 *Tak1*^*fl/fl*^ mice followed by 6 h or 72 h of reperfusion as previously described [33]. Briefly, anesthesia was induced with 3% of isoflurane in a 30% O_2_ balanced with 67% N_2_O and maintained in 1% isoflurane in 30% O_2_ and 69% N_2_O during surgery. To measure the regional cerebral blood flow (rCBF), a laser Doppler probe (Moor Instruments VMS-LDF2, Axminster, UK) was affixed to the skull above the left MCA territory after the left temporal bone between the ear and the eye. In supine position through a midline neck incision (< 1 cm), the left common carotid artery (CCA) and the external carotid artery (ECA) were isolated and ligated. For occlusion of MCA, a 2-mm silicon coated filament (Doccol, no. 701912PK5RE) was threaded into the internal carotid artery (ICA), and a sufficient occlusion was confirmed by reduction in rCBF to < 20% of the baseline value (supplemental Fig. 1D). Body temperature during surgery was maintained at 37 °C ± 0.5 °C using a feedback-controlled heating pad and a heating lamp. After 30 min of occlusion, mice were supplemented with 0.5 ml saline and placed into a temperature-controlled incubator before returning to their home cages with free access to food and water. Body weight and temperature were measured daily (Table 1). The sham group received the same surgical procedure, except the insertion of the silicon filament.

**Table 1:** Demographics and hematology after 30 min of tMCAo and 72 h of reperfusion

Demographics				
Genotype	*Tak1flf/l*	*Tak1flf/l*	*Cx3cr1creER-Tak1fl/fl*	*Cx3cr1creER-Tak1fl/fl*
Treatment	Corn oil	4HT	Corn oil	4HT
Age [weeks]	11 (± 1)	11 (± 1)	11 (± 1)	11 (± 1)
Bodyweight [g]	26.56 (± 2.31)	26.11 (± 2.53)	27.53 (± 2.76)	28.27 (± 2.64)
Weight loss (after 30 min of tMCAO and 72 h of reperfusion) [%]	15.80 (± 7.10)	10.81 (± 9.85)	11.40 (± 2.72)	13.25 (± 6.06)
Hematology				
WBC [103/μl]	4.40 (± 1.72)	5.92 (± 1.98)	5.23 (± 1.38)	4.067 (± 0.29)
RBC [106/μl]	9.56 (± 1.43)	9.52 (± 0.83)	9.36 (± 0.53)	10.12 (± 0.79)
HGB [g/dl]	14.30 (± 2.04)	14.07 (± 1.08)	13.80 (± 0.94)	14.60 (± 1.34)
HCT [%]	40.07 (± 5.86)	39.72 (± 2.91)	39.17 (± 2.54)	41.7 (± 3.28)
MVC [fl]	42.02 (± 1.21)	41.75 (± 0.68)	41.83 (± 1.31)	41.23 (± 0.45)
MCH [pg]	15.00 (± 0.43)	14.80 (± 0.22)	14.73 (± 0.66)	14.73 (± 0.66)
MCHC [g/dl]	35.70 (± 0.36)	35.40 (± 0.33)	35.20 (± 0.50)	34.97 (± 0.53)
PLT [10^3^/μl]	783.83 (± 268.01)	866.00 (± 196.61)	989.33 (± 187.65)	1033.00 (± 334.67)

*WBC*, white blood cells; *RBC*, red blood cells; *HGB*, haemoglobine; *HCT*, haematocrite; *MVC*, middle corpusculare volume; *MCH*, middle corpusculare hemoglobin; *MCHC*, middle corpusculare hemoglobin concentration; *PLT*, platelets. Full blood count without differential. Data are presented as mean ± SD (*n* = 6)

#### Exclusion criteria

Mice were excluded if a reduction in rCBF did not reach > 80% of the baseline value and if a recovery of rCBF to CCAo-level (60–70% of baseline) after 5 to 10 min reperfusion was lacking. In addition, animals with intracerebral hemorrhage, seizures, extensive weight loss (> 20% of starting weight), missing infarction in TTC-staining, and those that did not develop neurological deficits (mNSS < 5) were excluded. Mice that died during the observations period were excluded from all analyses, except mortality rate between genotypes and treatment (supplemental Fig. 1C). A scheme of treatment is given in supplemental Fig. 1B. All scorings and data acquisition were performed by blinded investigators.

### Neurological outcome

For evaluating the general status and focal neurologic dysfunction after tMCAo, a modified neurologic severity score (mNSS) as previously described by Habib et al. was used (Table 2) [33]. Three trials per test were performed by two individual investigators at 1 h before and 1 h, 6 h, 24 h, 48 h, and 72 h after tMCAo or sham surgery. The score ranges from 0 (no deficits) to 15 points representing the poorest performance in all items and is calculated as the sum of the general and focal deficits. For the inability to perform the task, abnormal performance, or for lack of a tested reflex one point, was awarded.

**Table 2:** Neurological assessment by 15-point modified neurologic severity score. Modified neurologic severity score used for evaluation of neurological dysfunction after tMCAo or sham surgery in a time-dependent manner following a 15-point scheme

Modified neurologic severity score	
behavioral test	
1. Flexion of forelimb	
2. Flexion of the hindlimb	
3. Cage grasp	
4. Head moving more than 10° to the vertical axis	
5. Immobility and staring	
6. Inability to walk straight	
7.Circling towards the paretic side	
8.Falling down to paretic side	
9. Climbing on 45° inclined surface	
10. Tremor	
11. Visual placing (suspended by the tail and slowly advanced toward a ledge, arch their back, and reach out with both forepaws 0P)	
12. Wisker response to light touch (1P when missing)	
13. Pinna reflex (head shake when touching auditory meatus)	
14. Corneal reflex (lightly touching the cornea with cotton, blink missing 1P)	
15. Startle reflex (motor response to a brief loud noise)	

### Infarct sizes and hematology

The infarct volume was measured using the 2,3,5-triphenyltetrazolium chloride (TTC) staining method as described earlier [33]. Six hours or 72 h after tMCAo or sham surgery, mice were deeply anesthetized, and EDTA blood was taken for a small blood count analysis in the Institute for Experimental Animal Science and Central Laboratory for Experimental Animals (RWTH Aachen) (Table 1). The brain was removed immediately and coronally sliced (1 mm), followed by short incubation at − 80 °C (for 1 min). After that, the brain sections were incubated in 2% TTC (Sigma Aldrich) for 10 min at 37 °C followed by a fixation in 10% formaldehyde. The stained sections were photographed and evaluated in a blinded manner using ImageJ software (NIH, Bethesda, MD., USA). The infarct volume was corrected for brain edema using the Reglodi’s method: Adjusted-lesion size = measured lesion × (contralateral hemisphere/ipsilateral hemisphere). Total infarct volumes were calculated by adding the mean area of each section and multiplied by the thickness of the sections.

### Reverse-transcription quantitative PCR (RT-qPCR)

Gene expression analyses were performed with tissue from the peri-infarct zone and the corresponding contralateral hemisphere using a stereomicroscopic approach. The tissue was immediately dissolved and homogenized in PeqGold (PeqLab, Germany). In the next step, total RNA was extracted using the peqGold RNA TriFast as previously described [33]. The RNA concentration was measured with a NanoDrop 1000 device (PeqLab). Complementary DNA was synthesized with the MMLV reverse transcription kit and random hexanucleotide primers (Invitrogen, Germany) using 1 μg/ml of total RNA. Quantitative reverse transcription PCR (RT-qPCR) analysis was performed using the MyIQ RT-qPCR detection system (Biorad, Germany). The target genes and one housekeeping gene [hypoxanthine guanine phosphoribosyltransferase (*hprt*)] were measured at cycle threshold (Ct values), and relative quantities were calculated by the ΔΔCt method using the qbase+ software (Biogazelle, Belgium). Data are expressed as relative amount of the target genes to *hprt*, respectively. A list of used primers including base pairs, sequences, and annealing temperature is given in Table 3. *Map3k7* primer targets the deleted exon 2 and exon 3 (for CGGAAGAGGAGCTTTTGGAGT; rev GGTTCACACGTGACAACTGC).

**Table 3:** Primers for RT-qPCR. Numbers of basepairs (bp) and sequences, annealing temperature, and species are shown

Target gene	Forward	Reverse	bp	Annealing temperature (°C)	Species
*hprt*	GCTGGTGAAAAGGACCTCT	CACAGGACTAGAACACCTGC	249	249	Mouse
*il1b*	GCACTACAGGCTCCGAGATGAAC	TTGTCGTTGCTTGGTTCTCCTTGT	147	61	Mouse
*tnf*	ATGGCCTCCCTCTCATCGT	CTTGGTGGTTTGCTACGACG	250	60	Mouse
*Il6*	GATACCACTCCCAACAGACCTG	GGTACTCCAGAAGACCAGAGGA	250	64	Mouse
*Map3k7*	CGGAAGAGGAGCTTTTGGAGT	GGTTCACACGTGACAACTGC	131	59	Mouse

### Western blot

Tissue from the ipsilateral and contralateral hemisphere or glial cell cultures which were lysed in ice-cold radioimmunoprecipitation assay buffer (RIPA) dosed with a protease and phosphatase inhibitor cocktail (Thermo Fischer Scientific, USA) were used for immunoblotting. Protein concentration was measured with a BCA-kit (Thermo Fischer Scientific, USA). We performed SDS-PAGE under denaturing and reducing conditions with 20–50 μg of the protein samples. That was followed by the blotting-transfer on a polyvinylidene difluoride (PVDF) membrane. Unspecific binding sites were blocked by incubation with 5% dry milk solved in 0.05% Tris-buffered saline (TBS) or 5% bovine serum albumin (BSA) for 1 h at room temperature. Primary antibody incubation was carried out in 5% dry milk/TBS or 5% BSA at 4 °C overnight. An appropriate secondary antibody in 5% dry milk/TBS was applied for 2 h at room temperature on the next day. Immunoreactivity was visualized by enhanced chemiluminescence (ECL Plus, Thermo Fischer Scientific, USA). β-Actin served as loading control. Densitometric evaluation was performed using ImageJ (NIH, Bethesda, MD., USA). A list of antibodies and dilutions is given in Table 4.

**Table 4:** Antibodies for western blot and immunocytochemical analyses. Manufacturer, catalog number, and dilution are given

Antibody	Manufacturer	Catalog number	Dilution
Western blot:			
Aktin	Santa Cruz	sc-47778	1:5000
Phospho-ERK1/2	Cell signalling	9101	1:1000
Phospho-SAPK/JNK	Cell signalling	4.67E+03	1:1000
Phospho-TAK1	Cell signalling	9339	1:1000
Phospho-p38	Cell signalling	9211	1:1000
Interleukin-1-β	Novusbio	NBP1-42767	1:1000
TNF-α	Novusbio	NBP1-19532	1:500
Immuncytochemistry:			
TAK1	Abcam	EPR5984	1:1000
GFAP	Santa Cruz	sc-33673	1:100
Iba-1	Millipore	MABN 92	1:200
Secondary antibodies:			
Goat anti-rabbit igG	BIO-RAD	170-6515	1:5000
Anti-mouse igG	SIGMA	A4416	1:4000
Donkey anti-rabbit IgG	Invitrogen	A21207	1:500
Donkey anti-goat IgG	Invitrogen	A11058	1:500

### Enzyme-linked immunosorbent assay (ELISA)

Enzyme-linked immunosorbent assay (ELISA) was performed according to manufacturers’ protocols (IL-1β Mouse Uncoated ELISA Kit Invitrogen; TNF-α ELISA Kit cell signalling). Plates were coated with the coating antibody and incubated at 4 °C overnight. Then, blocking was performed for 2 h at RT, followed by loading of the wells with 100 μl of each sample and 100 μl of an internal standard control at 4 °C overnight. Next, detection antibody was added for 2 h at RT followed by enzyme incubation (IL-1β Mouse Uncoated ELISA Kit: Avidin-HRP; TNF-α ELISA Kit: SAP). Last, substrate was added, and the plate was measured at 450 nm in the Tecan reader (Tecan GmbH, Switzerland).

### Primary murine cortical cell culture, oxygen glucose deprivation (OGD), and cell viability

Postnatal (P0 to P2) murine cortical cell culture preparation was performed as previously described [34]. In brief, after brain dissection meninges and blood vessels were removed, cortex was isolated, homogenized, and dissolved in Dulbecco’s phosphate-buffered saline (DPBS, Life Technologies, USA) containing 1% (v/v) trypsin and 0.02% (v/v) EDTA. The cell suspension was filtered through a 50-μm nylon mesh. After centrifugation (1400 rpm, 5 min), pellets were re-suspended in Gibco™ Dulbeccos’s modified Eagle medium (DMEM, Life Technologies, USA) and seeded on flasks in DMEM with additional 10% fetal bovine serum (FBS, PAA, Austria) and 0.5% penicillin-streptomycin (Invitrogen, USA). All flasks and plates were coated by poly-L-ornithine (PLO, Sigma-Aldrich, Germany) prior to cell seeding. Cells were kept in a humidified incubator at 37 °C and 5% CO_2_. For subcultivation, cells were trypsinized with 2.5% (v/v) trypsin diluted in PBS/EDTA and seeded on new flasks in a 1:3 ratio. In order not to lose non-adherent cells in the supernatant, we did not discard the cell supernatant during cultivation before trypsin but centrifuged it (5 min 1200 g). After decanting the supernatant, we resuspended the pellet of the non-adherent cells in the fresh medium and added it to the other cells. Medium was refreshed every second day. Subcultivation was performed when cells reached a confluence of about 80%. At passage 2, cells were seeded on experimental plates 48 h prior to stimulation.

OGD was performed in a self-constructed cube-shaped hypoxia chamber (28 × 14 × 26 cm). To induce severe hypoxia (< 1% O_2_), we flooded the hypoxic chamber with 100% nitrogen gas as previously described [33]. The RPMI medium which was used for hypoxia contained < 2 g/l glucose. OGD was performed for 3 h, and different reperfusion timepoints (1 h, 6 h, 72 h) were chosen for analysis. For analyzing the OGD-induced cell death, we determined the extent of necrosis and apoptosis by the release of the stable and soluble cytosolic enzyme lactate dehydrogenase (LDH) indicating leakage of the outer cell membrane (CytoTox 96® Non-Radioactive Cytotoxicity Assay, Promega, Germany). The assay was used according to the manufacturer’s protocol. Fluorescence or absorption was measured using a microplate reader (Tecan GmbH, Switzerland). Lysed cells served as internal positive controls, and viable cells without treatment as negative controls. Before performing OGD and measuring the LDH release, cells were either treated with 4-hydroxy-tamoxifen (4HT; 1 μM) for 48 h, with the TAK1-specific inhibitor 5-7-Oxozeaenol (1 μM) for 1 h [30], or with dimethyl sulfoxide (DMSO) as solvent control.

### Flow cytometry

Brains were removed, mechanically disrupted, and filtered through a 70-μm cell strainer to obtain a single cell suspension. After centrifugation, the cell suspension was resuspended in 22% Percoll gradient buffer and overlaid with cold DPBS. The gradient was centrifuged with a reduced acceleration and no brakes. Fluorescence-activated cell sorting was performed using the following gating strategy: cells were gated on the right size (SSC-A), granularity (FSC-A), and living cells (7-AAD vs. FSC). Exclusion of peripheral monocytes was achieved by Ly6C positivity (microglia are Ly6C negative). Cell doublets were excluded by FSC-A vs. FSC-H. At last, cells were gated on the microglia specific CD11b^high^ and CD45^lo^ (supplemental Fig. 2).

### Immunocytochemistry

After removal of the medium, cells were fixed with 3.7% PFA in PBS for 30 min at RT and washed three times with PBS. Then, cells were permeabilized by incubation with 0.2% Triton X-100 in PBS for 10 min at RT, followed by a blocking step with IFF buffer for 1 h. The primary antibody diluted in PBS was applied and incubated overnight at 4 °C. The following day, the second antibody diluted in PBS was applied for 1 h at RT. In addition, cell nuclei were stained with 4′,6-Diamidin-2-phenylindol (DAPI) (Roth, Karlsruhe, Germany).

Immunocytochemical staining was performed using antibodies against ionized calcium binding adaptor molecule 1 (IBA1) to detect microglia and against TAK1 (list of antibodies in Table 4). Results were evaluated with a Leica fluorescence microscope (Leica, Wetzlar, Germany).

### Immunohistochemistry and TdT-mediated dUTP-biotin nick end labeling (TUNEL) assay

Immunohistochemical TUNEL and DAPI staining were performed after tMCAo or sham surgery using “insitu cell death detection kit” (Rosch) and DAPI (Roth, Karlsruhe, Germany) according to the manufacturer’s protocol. After euthanasia, the brains were fixed with 4% paraformaldehyde (PFA) before paraffin-embedding process. Staining was performed on 5-μm sections of the “Bregma-0” region. First, the sections were deparaffinated and incubated for 10 min with citrate buffer in a microwave. Then, cells were permeabilized for 10 min in TBS containing 0.1% Triton X-100, followed by incubation with TUNEL reaction mix for 1 h at 37 °C in the dark and DAPI for 20 s. We counted apoptotic cells in twelve selected fields and expressed the number of apoptotic cells in the (peri-) infarct zone.

### Randomization, power- and statistical analysis

**Fig. 1:**
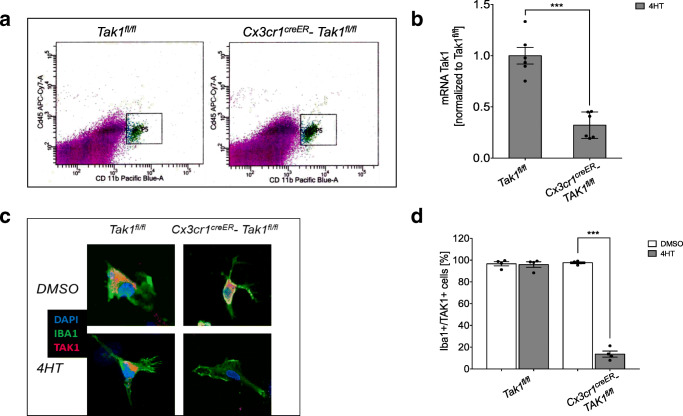
Microglial-specific TAK1-depletion was induced in *Cx3cr1*^*creER*^*-Tak1*^*fl/fl*^ mice after Tamoxifen-application. **a** Ex vivo-isolated microglia from *Tak1*^*fl/fl*^ and *Cx3cr1*^*creER*^*-Tak1*^*fl/fl*^ mice were characterized by surface expression of CD45^lo^ and CD11b 4 weeks after Tamoxifen application. **b***Map3k7*-mRNA level was measured in microglial cells by qRT-PCR of *n* = 6 mice. Data are presented as mean ± SD; individual data points are shown. Intergroup difference was tested by Students *t* test. **c** DAPI, IBA1, and TAK1 staining of representative microglial cells of *Tak1*^*fl/fl*^ and *Cx3cr1*^*creER*^*-Tak1*^*fl/fl*^ mice with different treatments and genotypes. **d** Cell counting of TAK1-positive microglia as the percentage of all counted microglia. Data are presented as mean ± SD; individual data points are shown. Intergroup difference was tested by ANOVA two-way

**Fig. 2:**
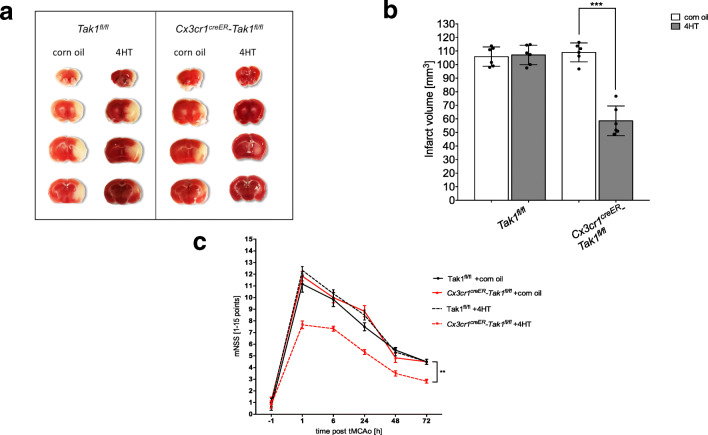
Microglial-specific TAK1-depletion reduced infarct volumes and neurological deficits after tMCAo. **a** Representative TTC stained brain sections of each treatment group and genotype. Necrotic tissue stained white. **b** Infarct volumes (measured by computer-assisted volumetry) of *Tak1*^*fl/fl*^ and *Cx3cr1*^*creER*^*-Tak1*^*fl/fl*^ mice with tamoxifen or vehicle treatment is shown (*n* = 6). Data are presented as mean ± SD; individual data points are shown. Intergroup differences were tested by ANOVA two-way. **c** Neurological assessment at different timepoints of *Tak1*^*fl/fl*^ and *Cx3cr1*^*creER*^*-Tak1*^*fl/fl*^ mice is demonstrated (*n* = 6)

**Fig. 3:**
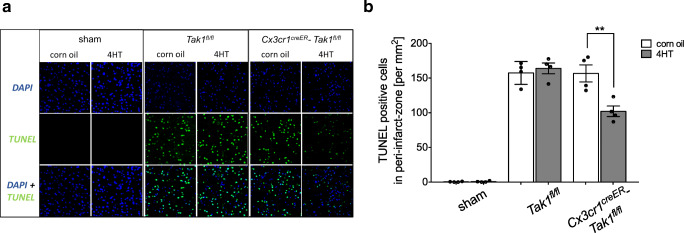
Microglial TAK1-depletion reduced apoptosis in the Peri-infarct-zone **a** TUNEL assay on paraffin-embedded coronal brain sections of mice sacrificed 72 h after 30 min of tMCAo. Pictures show representative extracts from peri-infarct-zone. Green fluorescence shows TUNEL positives cells. Blue fluorescence indicates DAPI staining of the nuclei. **b** Quantification of TUNEL-positive cells in peri-infarct zones. TUNEL-positive cells in six slices per mice (Bregma 0) with 12 × 0.01 mm^2^ grids per slice were counted. Bars represent means ± SD. *N* = 4 of every genotype and treatment group. Data are presented as mean ± SD; individual data points are shown. Intergroup differences were tested by ANOVA two-way

**Fig. 4:**
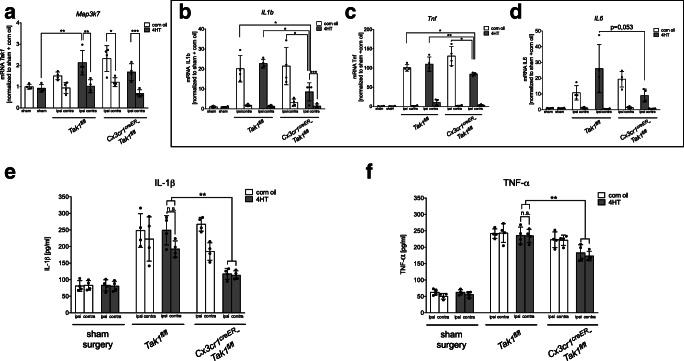
TAK1-depletion lowered the mRNA-expression and protein-level of pro-inflammatory IL-1β in the peri-infarct zone of ischemic brain tissue. **a** mRNA-levels of *Map3k7* in peri-infarct zone were measured by qRT-PCR after tMCAo (*n* = 4). **b**–**d** mRNA-levels of pro-inflammatory *il-1β*, *tnf-α*, and *il-6* in peri-infarct zone were measured by qRT-PCR after tMCAo (*n* = 4). **e**, **f** Protein-levels of IL-1β and TNF-α in peri-infarct zone were detected by ELISA (*n* = 4). All data are presented as mean ± SD; individual data points are shown. Intergroup differences were tested by ANOVA three-way

**Fig. 5:**
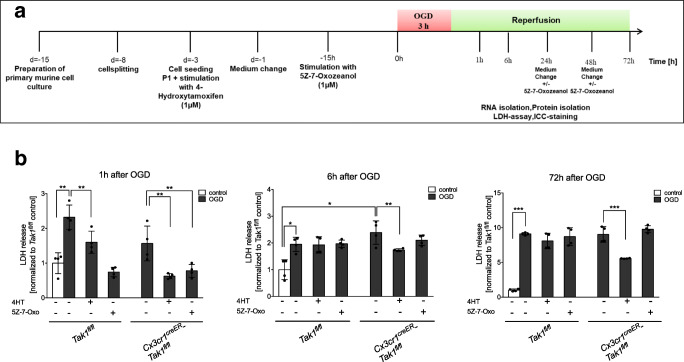
TAK1-inhibition reduced cell death rates in the first hour after OGD. **a** Schematic illustration of primary glial culture preparation, cell treatment, and OGD-procedure. **b** LDH-release in *Tak1*^*fl/fl*^ and *Cx3cr1*^*creER*^*-Tak1*^*fl/fl*^ glial cell cultures after 3 h of OGD followed by 1 h, 6 h, and 72 h of reperfusion is shown. Cells were treated with vehicle medium DMSO (“−”), Tamoxifen (4HT “+”) or with 5-7-Oxozeaenol (= 5-7-Oxo “+”) (*n* = 4). All data are presented as mean ± SD; individual data points are shown. Intergroup differences were tested by ANOVA two-way

**Fig. 6:**
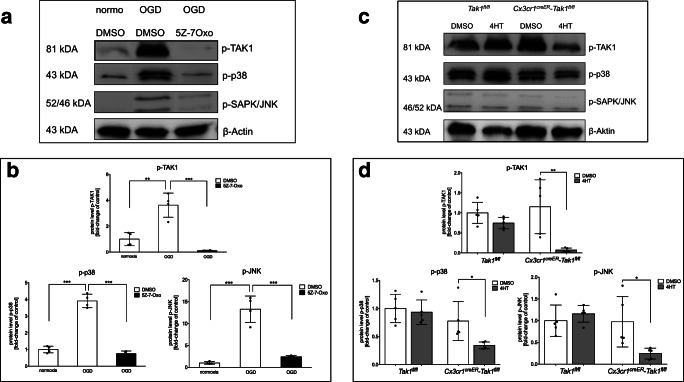
OGD-induced TAK1-activation results in p38- and JNK-phosphorylation in glial cells **a** Protein-levels of activated TAK1, p38, ERK1/2, and JNK were detected by immunoblotting directly after 3 h of OGD (*n* = 4). β-Actin served as a loading control. **b** Quantification of protein levels by densidometric analysis. **c** Protein-levels of activated TAK1, p38, ERK1/2, and JNK were detected by immunoblotting directly after 3 h of OGD (*n* = 4). β-Actin served as a loading control. **d** Quantification of protein-levels by densidometric analysis. normo, normoxia; 5Z-7-Oxo, 5Z-7-Oxozeaenol; DMSO, dimethylsulfoxide; 4HT, 4-Hydroxytamoxifen. All data are presented as mean ± SD; individual data points are shown. Intergroup differences were tested by ANOVA one-way (**b**) or two-way (**d**)

**Fig. 7:**
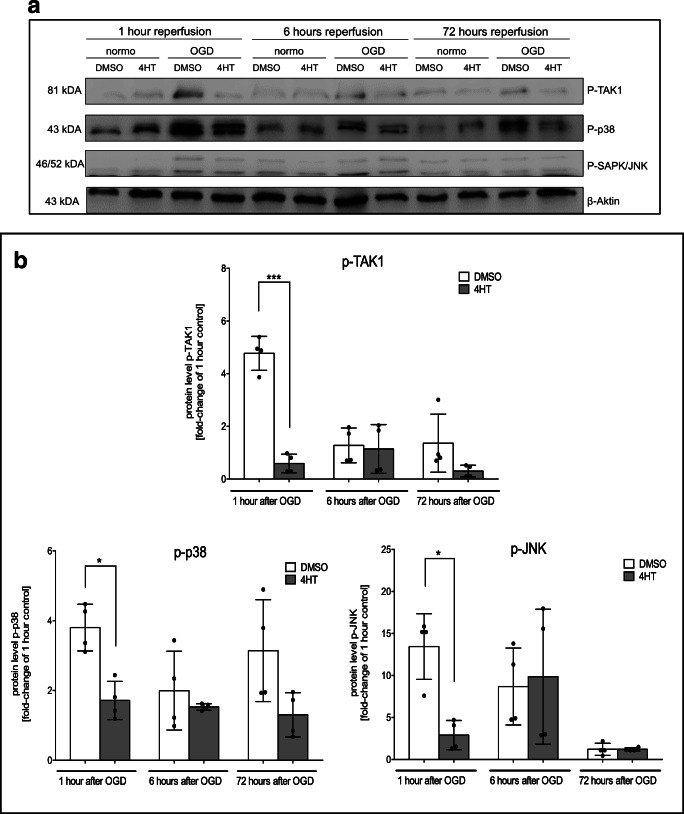
TAK1 activation occurs in early phase of oxygen-glucose-deprivation. **a** Protein-levels of activated TAK1, p38, ERK1/2, and JNK were detected by immunoblotting after 3 h of OGD and different timepoints after OGD *(n* = 4). β-Actin served as a loading control. **b** Quantification of protein levels by densidometric analysis. All data are presented as mean ± SD; individual data points are shown. Intergroup differences were tested by ANOVA three-way

All animals in this study were identified by earmark numbers and were randomly assigned to the treatment groups by a technical assistant not involved in the analyses. Randomization was carried out using sorting by random numbers (QuickCalcs, Graphpad prism 6.0). Surgeons were blinded for genotype and treatment of the mice, since pre- and post-operative genotyping and s.c. tamoxifen-injection (supplemental Fig. 1B) were performed by an independent technician. We assumed 25% difference in infarct volumes between *Tak1*^*fl/fl*^ mice and *Cx3cr1*^*creER*^*-Tak1f*^*l/fl*^ mice as neuroprotective and statistically significant. The standard deviation in the surgical procedure of tMCAo was approximately 15%. Since the 1st-order error (false positive) and the 2nd-order error (false negative) were assumed to be 5%, we required 10 animals per group. Power analysis was carried out with G*Power. The test for outliers was not conducted on the data. All statistical tests were performed using either JMP(R), Version 10. SAS Institute Inc., Cary, NC, 1989–2007 or GraphPad Prism version 8.2.1 for Windows, GraphPad Software, San Diego, California USA, www.graphpad.com. Prior to parametric testing of the data, residuals were analyzed for normal distribution using the Shapiro-Wilk normality test. Additionally, variance homogeneity was checked either by the Bartlett’s test for ANOVA one-way or Spearman’s test for heteroscedasticity for ANOVA two-way and three-way, respectively. When the normality test and/or test for variance homogeneity were significant, values were BOX-COX-transformed after calculation of the optimal lambda and used for statistical analysis according to the Handbook of Biological Statistics [35]. Intergroup differences were tested by Students *t* test (Fig. 1b), ANOVA one-way (Fig.6b), two-way (Figs. 1d, 2, 3, 5, and 6d), or three-way (Figs. 4 and 7) followed by Tukey post-hoc test (multiple groups). Data are given as arithmetic means ± SD. The level of significance was set as *p* ≤ 0.05 (=*). The individual data of each experiments is shown in the figures.

## Results

### Microglial-specific depletion of TAK1 resulted in reduced infarct sizes and attenuated neurological deficits after tMCAo

First, we aimed to demonstrate the microglial-specific depletion of TAK1 in *Cx3cr1*^*creER*^-*Tak1*^*fl/fl*^ mice after tamoxifen application. We sorted microglial cells of both genotypes by detecting surface expression of CD45^lo^ and CD11b^high^ 4 weeks after tamoxifen application (Fig. 1a). RT-qPCR analysis revealed more than 70% reduction of *Map3k7* mRNA in microglial cells of *Cx3cr1*^*creER*^*-Tak1*^*fl/fl*^ mice (mean ± SD = 0.29 ± 0.048, *p* < 0.0001) compared to the *Tak1*^*fl/fl*^ control group (Fig. 1b). On protein level co-immunocytochemical staining for IBA1- and TAK1-positivity (Fig. 1c) showed a depletion of cytoplasmatic expressed TAK1 in *Cx3cr1*^*creER*^*-Tak1*^*fl/fl*^ microglia after tamoxifen application (mean ± SD = 0.136 ± 0.028, *p* < 0.0001) (Fig. 1d).

We subjected a total of 37 *Tak1*^*fl/fl*^ and 37 *Cx3cr1*^*creER*^*-Tak1*^*fl/fl*^ 11-week-old male C57BL/6J mice in our preclinical randomized and blinded controlled trial to either 30 min of tMCAo or sham surgery followed by a reperfusion time of 6 h or 72 h. One animal (tamoxifen-treated *Tak1*^*fl/fl*^ mouse) died 3 h after surgery and two animals (tamoxifen-treated *Tak1*^*fl/fl*^ mouse and corn oil-treated *Cx3cr1*^*creER*^*-Tak1*^*fl/fl*^ mouse) were excluded from the study because of hemorrhages and extensive weight loss (> 20% of initial weight) (supplemental Fig. 1C). Laser Doppler flowmetry during every surgical procedure was used to assure sufficient MCA occlusion and revealed similar MCA-perfusion between genotypes (supplemental Fig. 1D). Both groups of mice revealed no significant differences in small blood count and weight loss after tMCAo (Table 1).

To examine infarct sizes, images of TTC stained brain slices were used after tMCAo (Fig. 2a). After 72 h of reperfusion, tamoxifen application had no influence on infarct volumes compared to corn oil application in the control group lacking cre-recombinase expression (105.89mm^3^ ± 7.89 mm^3^ vs. 108.52m^3^ ± 4.03 mm^3^). However, tamoxifen-induced microglial-specific TAK1-depletion in *Cx3cr1*^*creER*^*-Tak1*^*fl/fl*^ mice revealed a 50% reduction of lesion sizes compared to corn oil application and to the *Cre*^*ER*^-negative mice (mean ± SD = 58.4 mm^3^ ± 2.93 mm^3^, *p* < 0.0001) (Fig. 2b). In comparison, the animals subjected to 30 min of tMCAo followed by 6 h of reperfusion showed no significant differences of infarct sizes (supplemental Fig. 3).

Neurological examination was performed using a modified neurologic severity score (mNSS) to assess different aspects of neurologic functions as previously described (Table 2) [28]. In mice lacking microglial TAK1-expression, we observed statistically significant lower neurological deficits (e.g., less circling towards the paretic side and stronger cage grasp) compared to their vehicle-treated littermates and *Tak1*^*fl/fl*^ controls over the whole observation period of 72 h (5.4 ± 2.03, Fig. 2c).

To address the underlying mechanisms of the detected lower infarct sizes and better clinical outcome of *Cx3cr1*^*creER*^*-Tak1*^*fl/fl*^ mice after stroke, we further investigated the influence of microglial-specific TAK1 depletion on apoptosis and inflammation in tissue biopsies of the peri-infarct zone.

### Stroke-induced microglial TAK1 activation increases apoptosis and the level of pro-inflammatory cytokines in the peri-infarct zone

Cell death rates after ischemia were measured by TUNEL-assay in *Cx3cr1*^*creER*^*-Tak1*^*fl/fl*^ and *Tak1*^*fl/fl*^ mice 72 h after stroke (Fig. 3a). Compared to sham surgery, tMCAo eminently increased the number of TUNEL-positive cells in the ischemic brain regions (mean ± SD = 157.4 ± 8.25, *p* = <0.0001). We counted less apoptotic cells in the ipsilateral brain hemisphere of microglial TAK1-depleted mice (mean ± SD = 102.1 ± 7.67, *p* = 0.0093) compared to corn oil-treated control groups and the control group lacking Cre expression (Fig. 3b). Immunohistochemical double staining of TUNEL-positive cells with NeuN, Iba1, and GFAP revealed a predominantly neuronal apoptosis (supplemental Fig. 4).

To elucidate the effect of microglial TAK1 on neuroinflammation after stroke, brain biopsies from ipsilateral peri-infarct zone and the corresponding contralateral hemisphere (Bregma 0 ± 1 mm) were utilized for gene and protein expression analysis.

*Map3k7* expression was increased in the ipsilateral ischemic brain tissue compared to the contralateral brain tissue and to the sham control tissue (*p* < 0.01, Fig. 4a) and showed no significant differences between *Cre*^*ER*^-positive and *Cre*^*ER*^-negative mice. This was expected since *Map3k7* is expressed in all CNS cells including neurons, astrocytes, microglia, and oligodendrocytes [30]. Next, we observed an enhanced expression of *Il1b*, *Tnf*, and *Il6* in the ipsilateral brain hemisphere when compared to sham control and the contralateral hemisphere (Fig. 4b–d).

Microglial-specific TAK1-knockout significantly attenuated the increased *Il1b* and *Tnf* expression after ischemia (Fig. 4b, c; *IL1b*: *p* < 0.05; *Tnf*: *p* < 0.05). However, the depletion did not significantly change the level of *Il6* expression (Fig. 4D, *p* = 0.053).

These findings were further validated on protein level, using ELISA and Western Blot analysis to detect IL-1β and TNF-α. Both proteins were elevated after tMCAo when compared to the sham control in all groups (Fig. 4e, f; IL-1β: *p* = < 0.001; TNF-α: *p* = < 0.002). Notably, microglial TAK1-depletion reduced the ischemia-induced IL-1β and TNF-α protein level compared to the vehicle-treated control groups and the *Tak1*^*fl/fl*^ control group (Fig. 4e, f; *p* = < 0.001, *p* = < 0.005). Of note, in contrast to the ELISA, the Western Blot data did not show a significant effect of microglial TAK1-depletion on TNF-α levels (supplemental Fig. 5). The cytokine protein levels 6 h after tMCAo, however, did not reveal significant differences between *Cre*^*ER*^-positive and *Cre*^*ER*^-negative animals (supplemental Fig. 3).

In summary, microglial loss of TAK1 mitigates post-ischemic apoptosis and inflammation. To further verify the role of TAK1 after ischemia, we used OGD in glial cell cultures and compared pharmacological TAK1 inhibition (by 5Z-7-Oxozeaenol) with tamoxifen-induced microglial-specific TAK1 depletion.

### Global TAK1 inhibition alleviates cell death in the early phase, whereas microglial-specific TAK1 depletion displays prolonged cell protection

We subjected primary murine glial cells to 3 h of OGD as previously described [33] and measured cell death rates by detecting LDH-release after 1 h, 6 h, and 72 h of reperfusion time (Fig. 5a). A detailed figure including all corresponding controls is given in supplemental Fig. 6. First, 3 h of OGD induced cell death (up to tenfold after 72 h) compared to the normoxia control (Fig. 5b). Second, pharmacological TAK1-inhibtion by 5Z-7-Oxozeaenol as well as microglial TAK1-depletion dampened cell death rates 1 h after OGD. On the contrary, after prolonged reperfusion times (6 and 72 h after OGD), only microglial-specific TAK1-depleted cells (*p* < 0.005; *p* < 0.0005) maintained reduced LDH levels after OGD. The timely limited cytoprotective effect of general TAK1 inhibition highlights TAK1’s “dual sword” properties indicating that TAK1 might have positive regulatory functions on regeneration in other CNS cells than microglia.

### OGD-induced TAK1 activation results in p38 and JNK phosphorylation

Previous studies have shown that TAK1 activation occurs after brain injury [20, 29]. However, less is known about microglial TAK1 activation and downstream signalling as response to an ischemic stimulus. Thus, we first investigated the levels of activated/phosphorylated TAK1 and TAK1-dependent downstream signalling proteins JNK and p38 after 3 h of OGD by western blot analysis (Fig. 6a). OGD increased levels of phosphorylated TAK1 (*p* < 0.003), phosphorylated p38 (*p* < 0.0001), and phosphorylated JNK (*p* = 0.0002) compared to control conditions (Fig. 6b). We also measured levels of p-ERK as a TAK1-independent MAP kinase with potential to promote inflammatory signalling. However, levels of activated ERK were not changed by OGD (supplemental Fig. 7). The administration of the TAK1-inhibitor 5Z-7-Oxozeaenol extensively diminished levels of p-TAK1, p-p38, p-JNK after OGD, suggesting that activation of these required TAK1 as an activator after ischemia (Fig. 6b; *p* < 0.0005). P-ERK levels were also diminished by 5Z-7-Oxozeaenol. As expected, the microglial depletion of TAK1 lead to reduced levels of p-TAK1 (*p* < 0.008) coinciding with the reduced expression of TAK1 protein in microglia. Moreover, less p-p38 (*p* < 0.03) and less p-JNK (*p* < 0.03) were detected after OGD (Fig. 6c, d). In *Tak1*^*fl/fl*^ mice, tamoxifen treatment had no effect on the phosphorylation of the analyzed proteins.

### Highest level of TAK1 activation and phosphorylation of downstream kinases is detected in the first hour after OGD

In order to examine the timepoint of TAK1/MAPK activation, we measured p-TAK1, p-p38, p-JNK, and p-ERK levels in a time-dependent manner after OGD followed by 1 h, 6 h, and 72 h of reperfusion (Fig. 7a).

In line with data obtained directly after OGD (Fig. 6), TAK1, JNK, and p38 activation was eminent 1 h after ischemia (see supplemental Fig. 8; p-TAK1: *p* < 0.006; p-JNK: < 0.01; p-p38: < 0.01). After 6 h, levels of p-TAK1 remained elevated, but in line with amounts of p-JNK and p-p38 did not differ significantly from the control condition. Seventy-two hours after OGD, we observed equal levels of these phosphorylated proteins. Consistent with previous results, tamoxifen-induced microglial TAK1-depletion dampened the increasing levels of p-TAK1, p-p38, and p-JNK 1 h after ischemia (p-TAK1: *p* < 0.005; p-JNK: < 0.05; p-p38: < 0.05), which was diminished after 6 h. This suggests that TAK1-mediated inflammation and apoptosis are triggered in the early phase of ischemia.

## Discussion

Beneficial effects of TAK1 inhibition have already been observed in models of various neurological diseases. In most cases, a general TAK1 inhibition by 5Z-7-Oxozeaenol, a specific small molecule inhibitor, was used [36, 37]. However, limited conclusions can be drawn from these studies, since cell-specific TAK1 functions were often neglected [11, 14]. Microglia are crucial for the activation of inflammatory cascades and TAK1 is known as a central mediator of neuroinflammation after cerebral ischemia [38, 39]. In this study, we examined the role of TAK1 after cerebral ischemia and compared the impact of a pharmacological TAK1 inhibition and microglial-specific TAK1 depletion on post-ischemic neuroinflammation and apoptosis.

First, microglial TAK1 depletion was induced via tamoxifen-dependent conditional depletion in male mice. The cDNA of an estrogen-ligand-binding receptor was fused to *Cre* recombinase and expressed under control of the *Cx3cr1* promotor, which is constitutively expressed in macrophage-like cells [40–42] (see supplemental Fig. 1A). Verifying data by Goldman et al., we have been able to demonstrate that microglia lack the expression of TAK1 4 weeks after tamoxifen injection due to their concomitant self-renewal (Fig. 1). Macrophages and dendritic cells are completely replaced in this time and express TAK1 on a normal level [30]. However, recent studies [43–45] have shown that the B6J.B6N(Cg)-*Cx3cr1*^*tm1.1(cre)Jung*^/J line, which Goldman et al. and we also used, showed leakiness into neurons. Even though this model has a high specificity for microglial cells, it cannot be excluded that some of the effects occur via modulation of TAK1 in other cells including neurons. Zhao and colleagues emphasized that tamoxifen treatment, however, led to a broad expression of the reporter in microglial cells without significant leakage into neurons. Moreover, the authors suggest that a potential leakiness of the GFP reporter should be tested and included as a control in cell-tracing experiments [43].

In our randomized controlled and blinded trial, we demonstrated that microglial loss of TAK1 leads to a reduction of infarct volumes, decreased neurological deficits, and an attenuated apoptosis rate in the peri-infarct-zone 72 h after stroke. In contrast, injection of tamoxifen had no effect on these parameters in the *Tak1*^*fl/fl*^ control group, suggesting that the neuroprotective effect in our model is not mediated through tamoxifen application which was described by Metha et al. [46]. Six hours after stroke, no significant difference in infarct sizes was detectable. It is important to emphasize that in the current study, the young age of included mice (11 weeks) and the two timepoints of reperfusion have to be seen as limitations of our study, since it is well known that stroke is a disease of aged individuals and that microglia can change their activation and expression patterns in older age. The latter is described for aging microglia, which developed a phenotype that is more inflammatory and cytotoxic, and which created an inimical environment for neurons [47, 48]. However, microglial cells are also known to be involved in repair mechanism in the acute phase after ischemic stroke [49, 50]. For a better translation of animal models into clinical use, the aspect of age and longer reperfusion times (e.g., 7 days, 14 days) after tMCAo should be considered in future studies.

Gene expression analysis from biopsies of the peri-infarct zone revealed a reduction of *Il1b* and *Tnf* mRNA after microglial-specific TAK1 depletion. Protein levels of IL-1β and TNF-α were also reduced, strengthening the PCR data. It is known that cerebral ischemia leads to an activation of the inflammasome complex in microglia which results in maturation and secretion of IL-1β [39]. Therefore, it is not unreasonable to hypothesize that TAK1 might play an important role in ischemia-dependent activation of the inflammasome due to its regulating effect on *Il1b* mRNA/IL-1β protein maturation. In addition, TAK1 is also involved in the activation of the NF-KB transcription factor, which again controls, among others, the expression of the inflammasome component *Nlrp3* [17, 51]. Further studies should therefore address the impact of microglial TAK1 on expression of inflammasome components and inflammasome-dependent signalling proteins. Chauhan et al. [52] has recently been able to show that myeloid TAK1 depletion resulted in smaller infarct sizes and improved functional outcomes accompanied by a reduction of brain-infiltrating immune cells (primarily monocytes) after 3 and 7 days post MCAo. Whether TAK1 depletion in monocytes in our model might also exert a protective impact as well, cannot be completely excluded. However, Goldmann et al. [28] was able to demonstrate that 4 weeks after TAM application, almost exclusively microglial cells stably express the cre recombinase due to their longevity and their capacity of self-renewal, whereas the cre in monocytes were virtually undetectable. Therefore, the described results appear to be due to a microglial-specific TAK1 depletion.

Comparing microglial-specific TAK1 depletion with global pharmacological TAK1 inhibition after OGD, the latter dampened LDH release only in the first hour, whereas microglial-specific TAK1 depletion was cytoprotective over the whole observation period of 72 h. Neubert et al. have also described a protective effect of pharmacological TAK1 inhibition in neurons in the early phase after hypoxia [24]. The cell-specific knockout of TAK1 delivers a more promising approach, since the dual properties of TAK1 seem to be taken in account.

In addition, we showed a strong activation of TAK1 and its downstream signalling proteins p38 and JNK after hypoxic stimulus. While microglial depletion of TAK1 resulted in reduced levels of p-TAK1, p-p38, and p-JNK after OGD, 5Z-7 Oxozeaenol-treatment completely diminished phosphorylation levels of the aforementioned proteins. The paralleled reduction of p38 and JNK signalling after TAK1 inhibition and its impact on cell survival imply that these two kinases might be responsible for the neurotoxic effect of TAK1 activation after ischemia. Our results suggest that neither ischemia/OGD nor microglial-specific TAK1 depletion seems to have an impact on p-ERK levels after all time points studied (supplemental Fig. 7). Interestingly, however, 5-7-Oxo as a widespread TAK1 inhibitor also lowered p-ERK levels, which may indicate a general MAP kinase inhibition ability. A more detailed investigation of whether this is a dose-dependent effect or whether it generally occurs after 5-7-Oxo administration or whether other MAP kinases are also inhibited remains to be explored.

Furthermore, strongest activation of TAK1 (p-TAK1) and its downstream signalling proteins p-p38, p-JNK is seen shortly after OGD (1 h) but is attenuated after 6 h and completely diminished after 72 h of reperfusion. This highlights a potential involvement of MAP-kinase pathways in TAK1-mediated post-ischemic neuroinflammation and apoptosis for glial cells and especially for microglial cells. The fact that genetic depletion of TAK1 in neurons and the prolonged treatment with 5Z-7-Oxozeaenol did not confer protection against cerebral ischemia [24] indicates that TAK1-mediated inflammation is mainly triggered in other brain cells than neurons. The lacking effect of prolonged 5Z-7-Oxozeaenol treatment could be explained by an upregulation of other MAP3Ks such as ASK1 [24]. Especially the microglial-specific inhibition of TAK1 seems to dampen post-ischemic neuroinflammation sustainably and improve neurological outcome after stroke indicating that microglia exert detrimental pro-inflammatory and pro-apoptotic effects in the early phase after ischemia. These findings might deliver further information regarding the role of microglial cells in stroke pathophysiology.

## Electronic supplementary material

Supplemental Figure 1 **Intraoperative laser doppler flowmetry revealed no differences between genotypes (A)** Illustration of the tamoxifen-induced knockout model. **(B)** Schematic illustration of the treatment protocol, weight measurement and neurological assessment. **(C)** Scheme of protocol summarizing the number of total animals (74 mice), with exclusion (excl.) per group and included animal (incl.) for final analysis. “†” indicates dead animals. Exclusion criteria are described in material and methods section. **(D)** Ipsilateral Laser Doppler flowmetry changes by intraluminal MCAO procedure were monitored. The baseline blood flow was considered as 100% for all mice. Change in LDF after occlusion of CCA, MCA and reperfusion are demonstrated. Bars represent means ± SD. *Abbreviations.* bp: basepairs*;* CCAo: Common Carotid Artery occlusion; MCAo: Middle Cerebral Artery occlusion; LDF: Laser Doppler flowmetry. (PDF 431 kb)

Supplemental figure 2 **Flow cytometry gating strategy for microglial cells. Analytic gating of flow cytometry data: (A)** Microglia were gated on the right size (SSC-A (size) vs. FSC-A (granularity)). **(B)** Doublets were excluded with FSC-A vs. FSC-H. **(C)** Gating on the living cells was performed (7-AAD vs. FSC). **(D)** Peripheral monocytes were excluded by gating Ly6Cpos cells (SSC vs. Ly6C)**. (E)** Microglial specific surface markers CD11bhigh and CD45int were used for gating. (PDF 6593 kb)

Supplemental figure 3 **Microglial-specific TAK1-depletion reveals no differences of infarct volumes and inflammatory cytokines after 30 min of tMCAo followed by 6 h of reperfusion**. (A) Representative TTC stained brain sections of each treatment group and genotype are shown. Necrotic tissue is stained white. (B) Infarct volumes of Tak1fl/fl and Cx3cr1creER-Tak1fl/fl mice with tamoxifen treatment is shown (*n* = 4), Mann-Whitney U test. (C,D). IL-1β and TNF-⍺ levels of ipsilateral and contralateral brain tissue after 30 min of tMCAo followed by 6 h of reperfusion and the sham controls were measured by ELISA. Data are presented as mean ± SD. Intergroup differences were tested by ANOVA two-way. (PDF 321 kb)

Supplemental figure 4 **TUNEL / NeuN costaining reveals predominantly neuronal apoptosis in the peri infarct area. (A**) Double staining of TUNEL (green) and NeuN (red); nuclei were counterstained with DAPI (blue). TUNEL-positive cells mainly colocalized with neurons. High numbers of apoptotic cells were observed in tMCAo groups. **(B)** Double staining of TUNEL (green) and Iba1 (red); nuclei were counterstained with DAPI (blue). High numbers of apoptotic cells were observed in tMCAo groups. **(C)** Double staining of TUNEL (green) and GFAP (red); nuclei were counterstained with DAPI (blue). High numbers of apoptotic cells were observed in tMCAo groups. (PDF 1422 kb)

Supplemental Figure 5 **Western Blot analysis of pro-inflammatory IL-1β and TNF-α in peri-infarct zone 72 h after tMCAo. (A,B)** Protein-levels of IL-1β and TNF-α in peri-infarct zone were detected by immunoblotting (*n* = 6). β-Actin served as loading control. Quantification of protein-levels by densidometric analysis. All data are presented as Mean ± SD, individual data points are shown. Intergroup differences were tested by ANOVA three-way. (PDF 353 kb)

Supplemental Figure 6 **TAK1-Inhibition reduced cell death rates in the first hour after OGD. (A)** Schematic illustration of primary glial culture preparation, cell treatment and OGD-procedure. **(B)** LDH-release in *Tak1*^*fl/fl*^ and *Cx3cr1*^*creER*^*-Tak1*^*fl/fl*^ glial cell cultures after 3 h of OGD followed by 1 h, 6 h and 72 h of reperfusion is shown. Cells were treated with vehicle medium DMSO (“-”), Tamoxifen (4HT “+”) or with 5-7-Oxozeaenol (=5-7-Oxo “+”) (n = 4). All data are presented as Mean ± SD, individual data points are shown. Intergroup differences were tested by ANOVA two-way. (PDF 144 kb)

Supplemental figure 7 **Glial P-ERK levels do not change in response to OGD in mixed glial cell cultures. (A,B,C)** Protein levels of activated ERK1/2 were detected by immunoblotting after 3 h of OGD and different timepoints after OGD (n = 4). β-Actin served as a loading control. Quantification of protein levels by densidometric analysis. (PDF 1471 kb)

Supplemental figure 8 **TAK1 activation occurs in early phase of Oxygen-glucose-deprivation. (A)** Protein levels of activated TAK1, p38, ERK1/2, JNK were detected by immunoblotting after 3 h of OGD and different timepoints after OGD (n = 4). β-Actin served as a loading control. **(B)** Quantification of protein levels by densidometric analysis. All data are presented as Mean ± SD, individual data points are shown. Intergroup differences were tested by ANOVA three-way. (PDF 603 kb)

Supplemental figure 9 Immunocytochemical staining of primary murine cortical cell culture of six independent preparations revealed 90.1% (± 3.3%) astrocytes, 6.3% (± 1.84%) microglia and 3% (± 1.7%) oligodendrocytes were found. We did not detect NeuN positive cells in our P2 cells. (PDF 36 kb)
