# Supplementary figures and images for: Microglial-specific depletion of TAK1 is neuroprotective in the acute phase after ischemic stroke
Source: J Mol Med (Berl). 2020 May 7;98(6):833–47. doi: 10.1007/s00109-020-01916-9 (PMC7297861; doi:10.1007/s00109-020-01916-9)

**A**

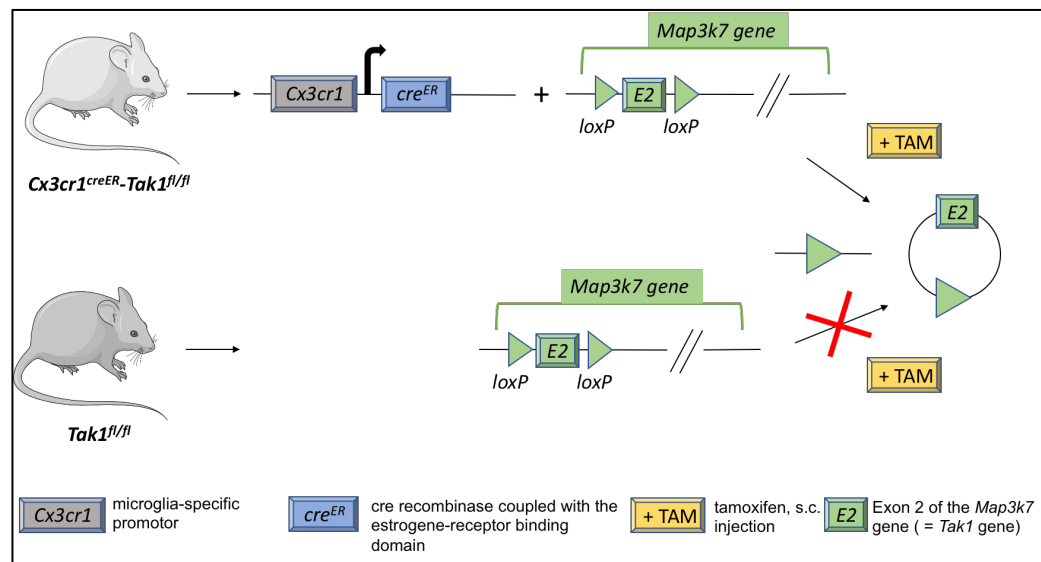

**B**

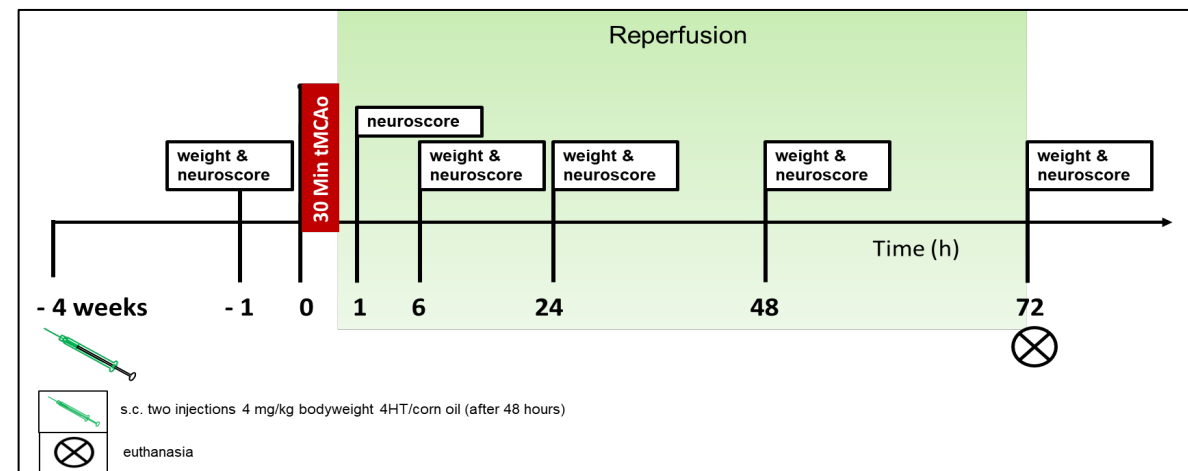

**C**

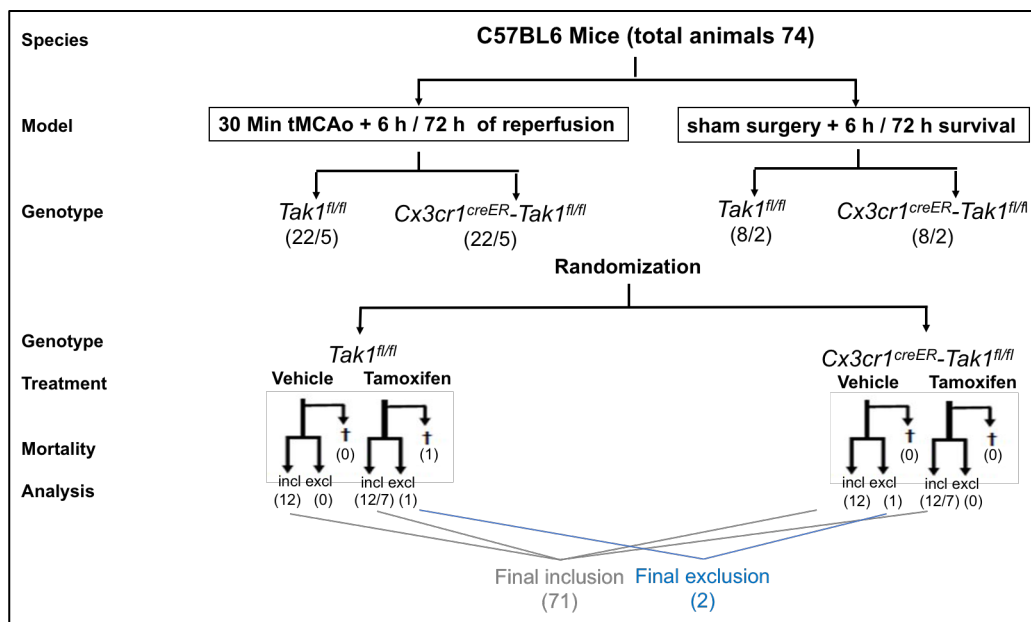

**D**

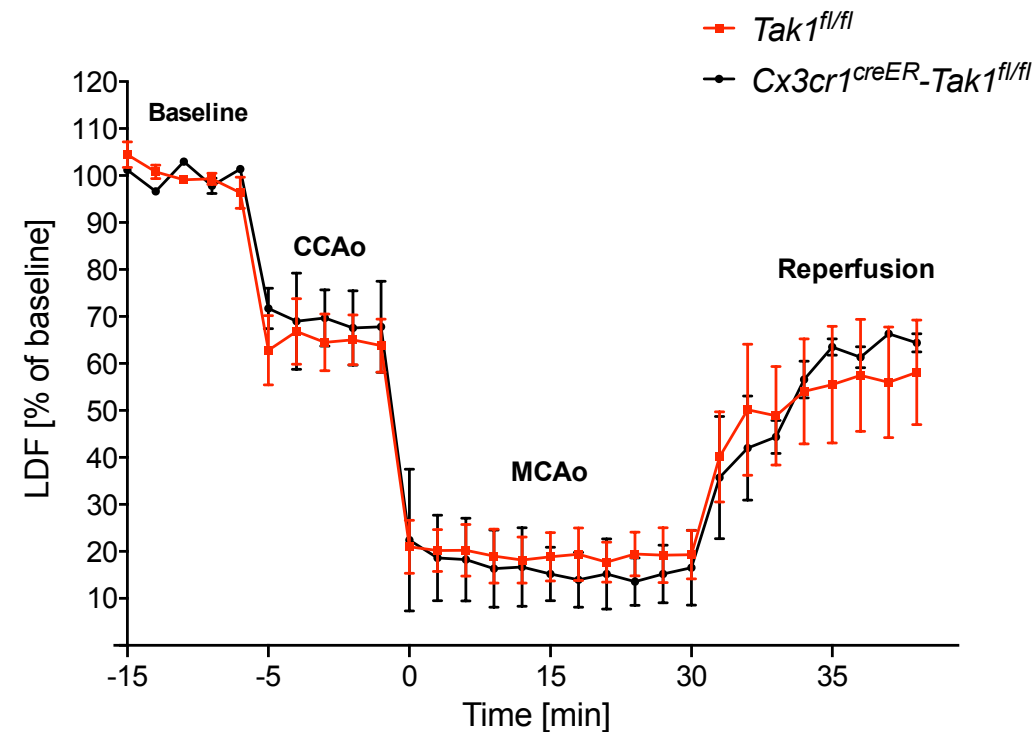

Supplement: Supplementary file 1 — Intraoperative laser doppler flowmetry revealed no differences between genotypes (A) Illustration of the tamoxifen-induced knockout model. (B) Schematic illustration of the treatment protocol, weight measurement and neurological assessment. (C) Scheme of protocol summarizing the number of total animals (74 mice), with exclusion (excl.) per group and included animal (incl.) for final analysis. “†” indicates dead animals. Exclusion criteria are described in material and methods section. (D) Ipsilateral Laser Doppler flowmetry changes by intraluminal MCAO procedure were monitored. The baseline blood flow was considered as 100% for all mice. Change in LDF after occlusion of CCA, MCA and reperfusion are demonstrated. Bars represent means ± SD. Abbreviations. bp: basepairs; CCAo: Common Carotid Artery occlusion; MCAo: Middle Cerebral Artery occlusion; LDF: Laser Doppler flowmetry. (PDF 431 kb) [file 109_2020_1916_MOESM1_ESM.pdf]

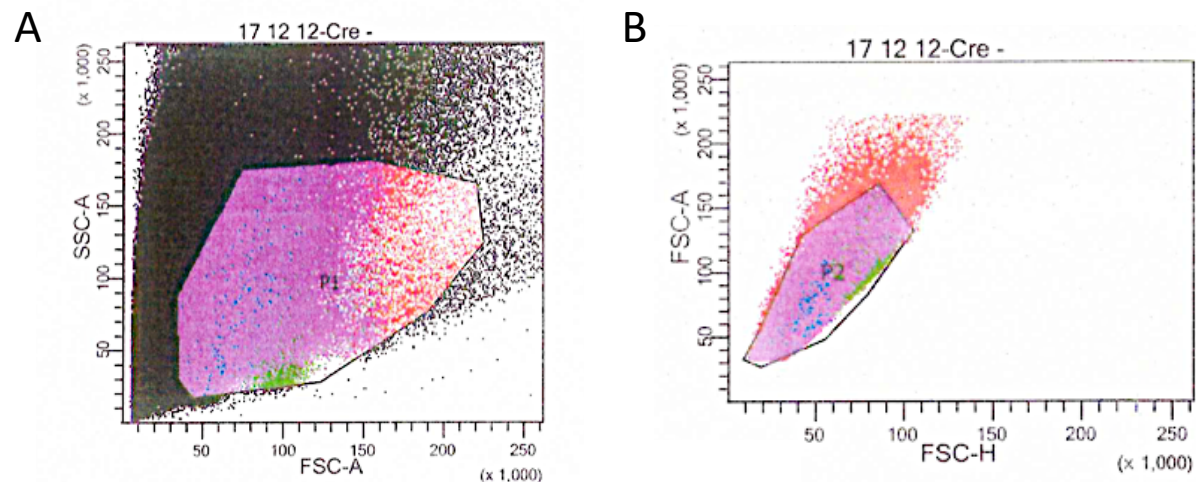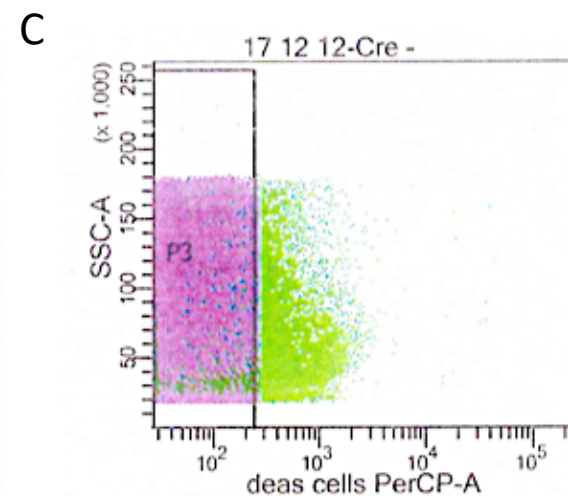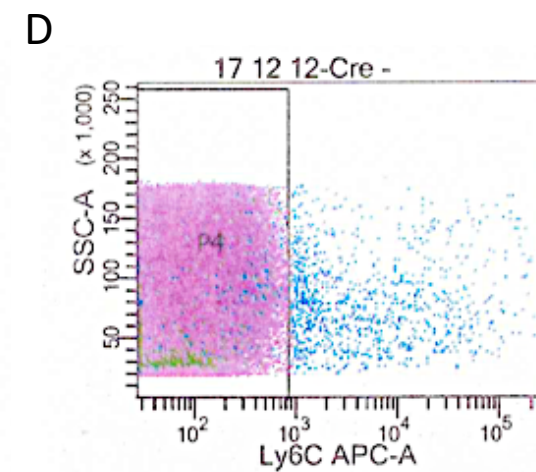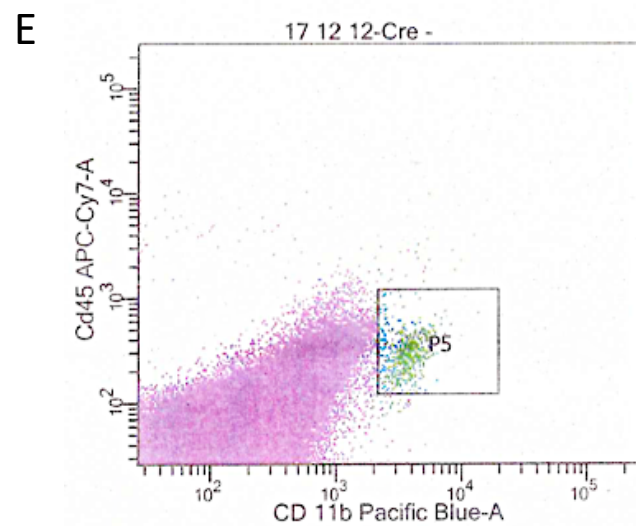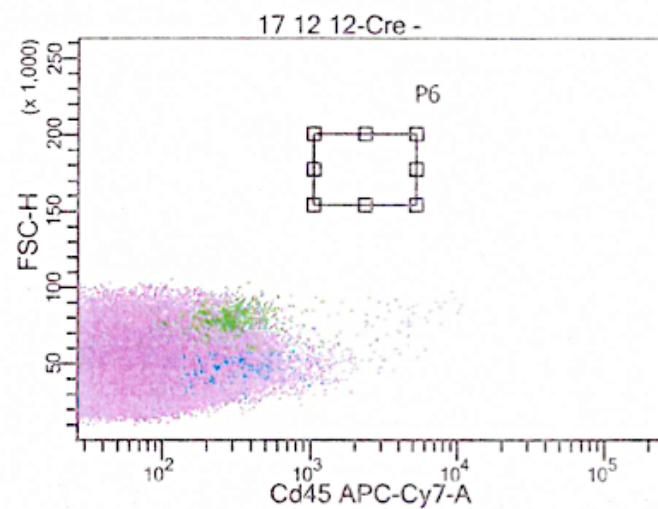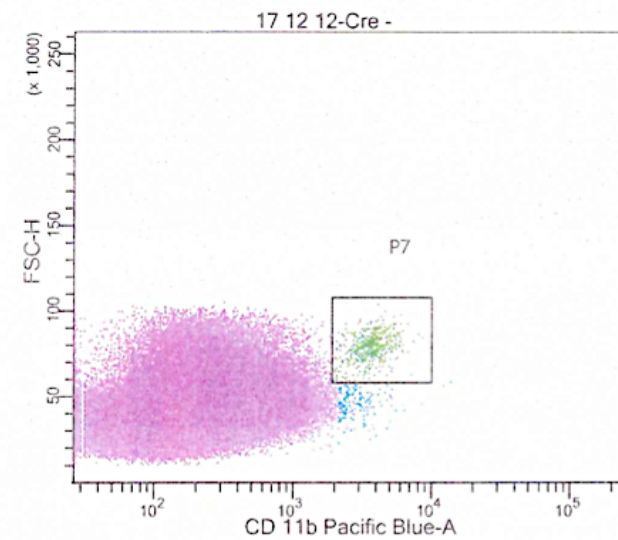

Supplement: Supplementary file 2 — Flow cytometry gating strategy for microglial cells. Analytic gating of flow cytometry data: (A) Microglia were gated on the right size (SSC-A (size) vs. FSC-A (granularity)). (B) Doublets were excluded with FSC-A vs. FSC-H. (C) Gating on the living cells was performed (7-AAD vs. FSC). (D) Peripheral monocytes were excluded by gating Ly6Cpos cells (SSC vs. Ly6C). (E) Microglial specific surface markers CD11bhigh and CD45int were used for gating. (PDF 6593 kb) [file 109_2020_1916_MOESM2_ESM.pdf]

**A**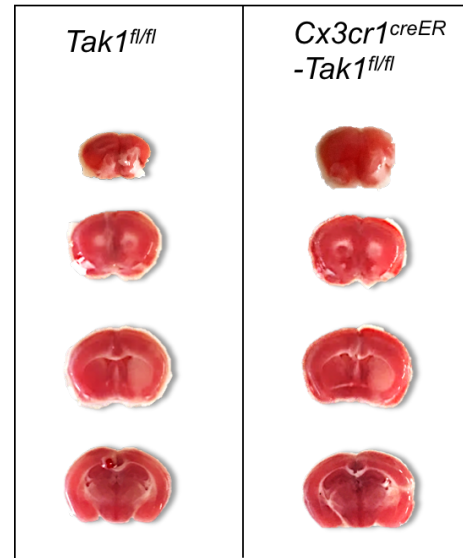**B**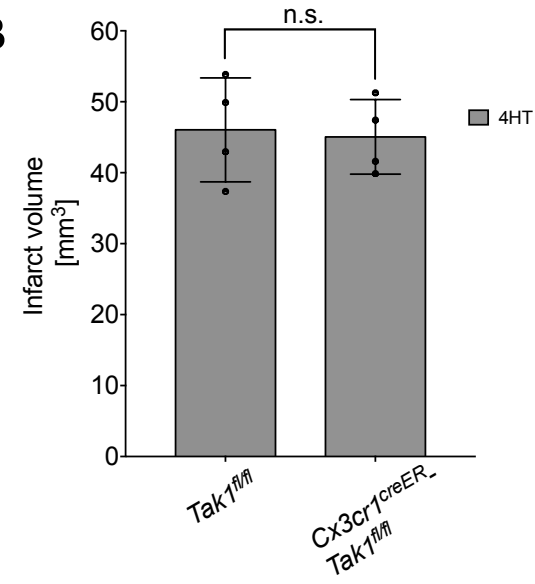**C**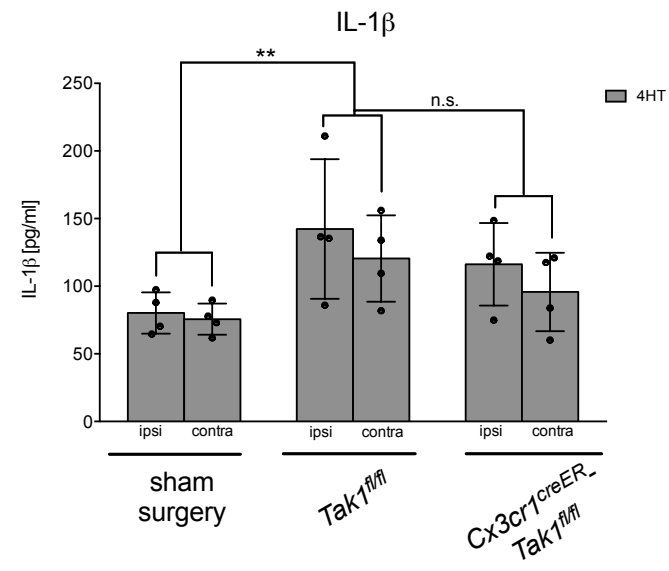**D**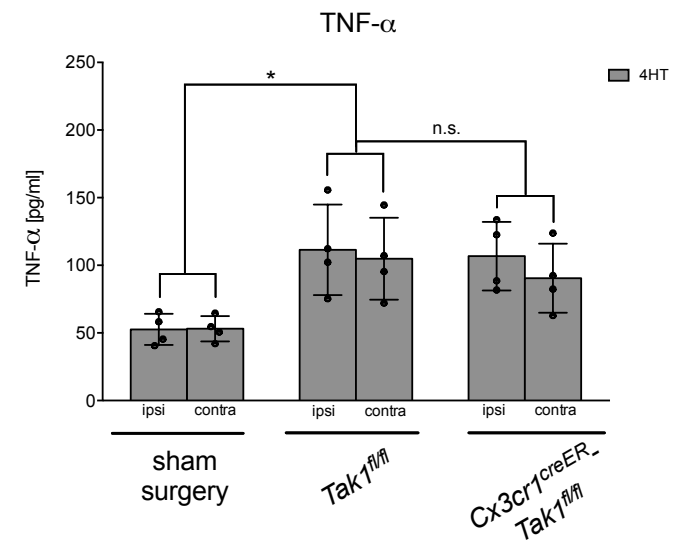

Supplement: Supplementary file 3 — Microglial-specific TAK1-depletion reveals no differences of infarct volumes and inflammatory cytokines after 30 min of tMCAo followed by 6 h of reperfusion. (A) Representative TTC stained brain sections of each treatment group and genotype are shown. Necrotic tissue is stained white. (B) Infarct volumes of Tak1fl/fl and Cx3cr1creER-Tak1fl/fl mice with tamoxifen treatment is shown (n = 4), Mann-Whitney U test. (C,D). IL-1β and TNF-⍺ levels of ipsilateral and contralateral brain tissue after 30 min of tMCAo followed by 6 h of reperfusion and the sham controls were measured by ELISA. Data are presented as mean ± SD. Intergroup differences were tested by ANOVA two-way. (PDF 321 kb) [file 109_2020_1916_MOESM3_ESM.pdf]

A

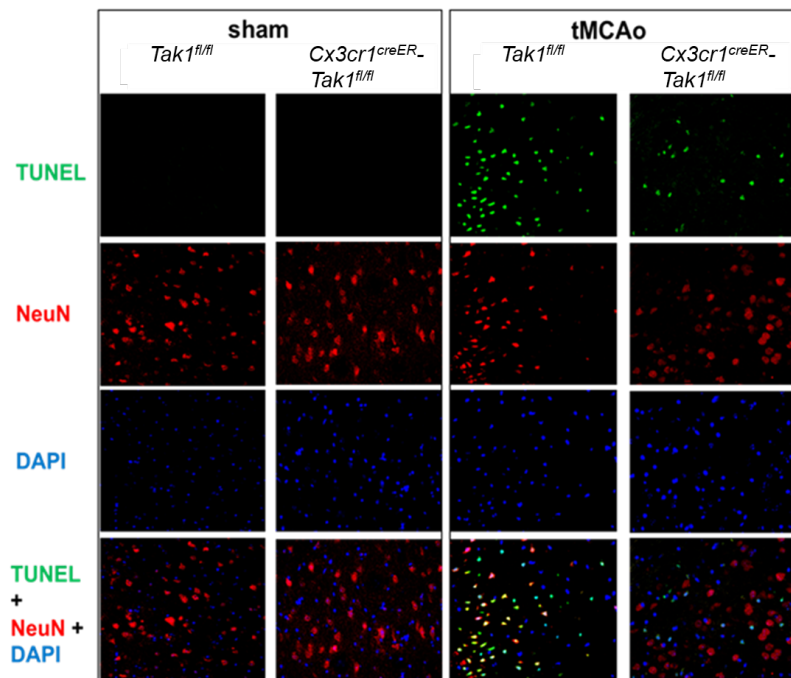

NeuN

B

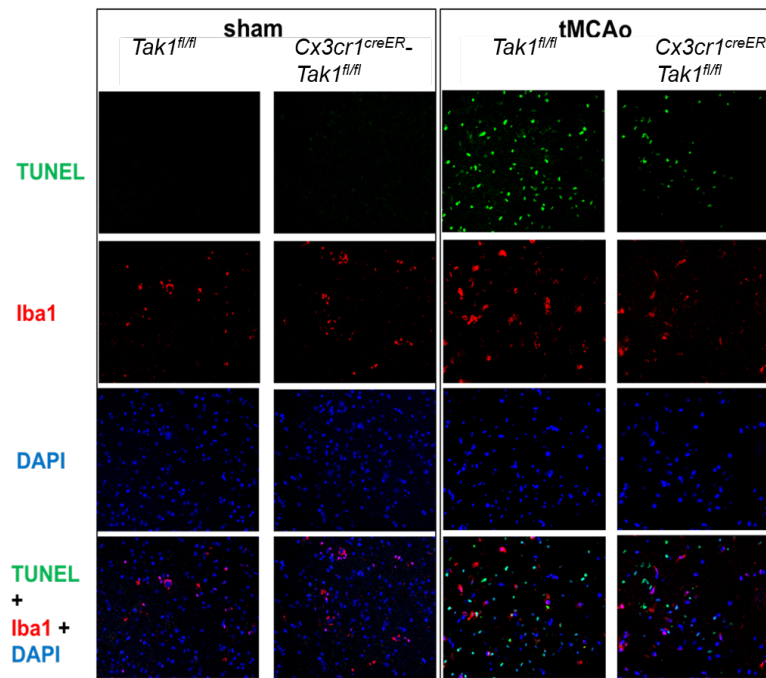

Iba1

C

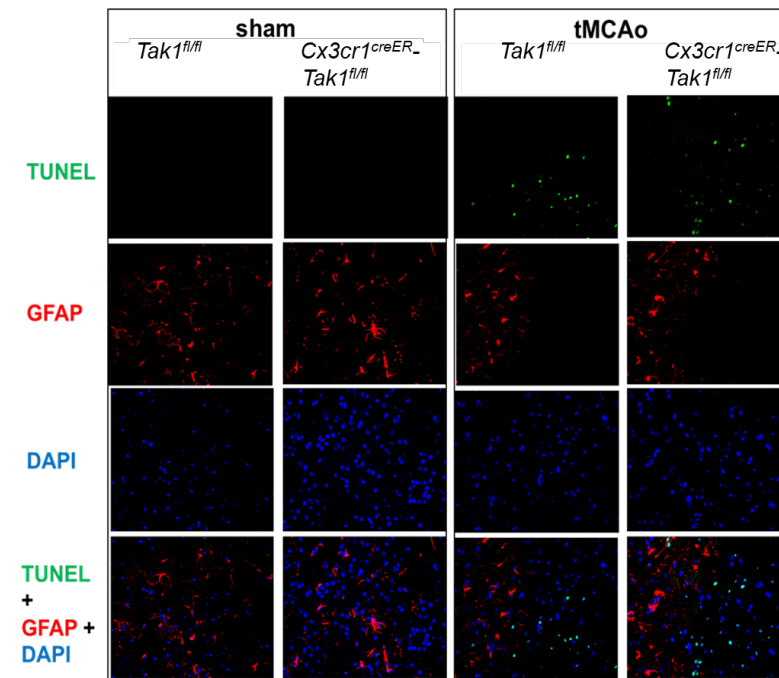

GFAP

Supplement: Supplementary file 4 — TUNEL / NeuN costaining reveals predominantly neuronal apoptosis in the peri infarct area. (A) Double staining of TUNEL (green) and NeuN (red); nuclei were counterstained with DAPI (blue). TUNEL-positive cells mainly colocalized with neurons. High numbers of apoptotic cells were observed in tMCAo groups. (B) Double staining of TUNEL (green) and Iba1 (red); nuclei were counterstained with DAPI (blue). High numbers of apoptotic cells were observed in tMCAo groups. (C) Double staining of TUNEL (green) and GFAP (red); nuclei were counterstained with DAPI (blue). High numbers of apoptotic cells were observed in tMCAo groups. (PDF 1422 kb) [file 109_2020_1916_MOESM4_ESM.pdf]

A

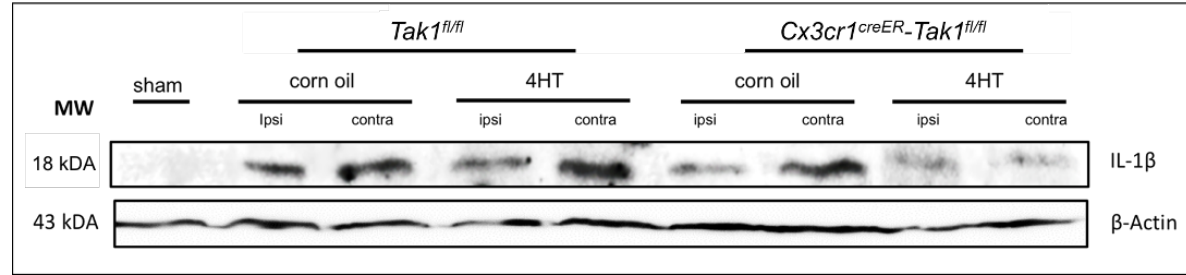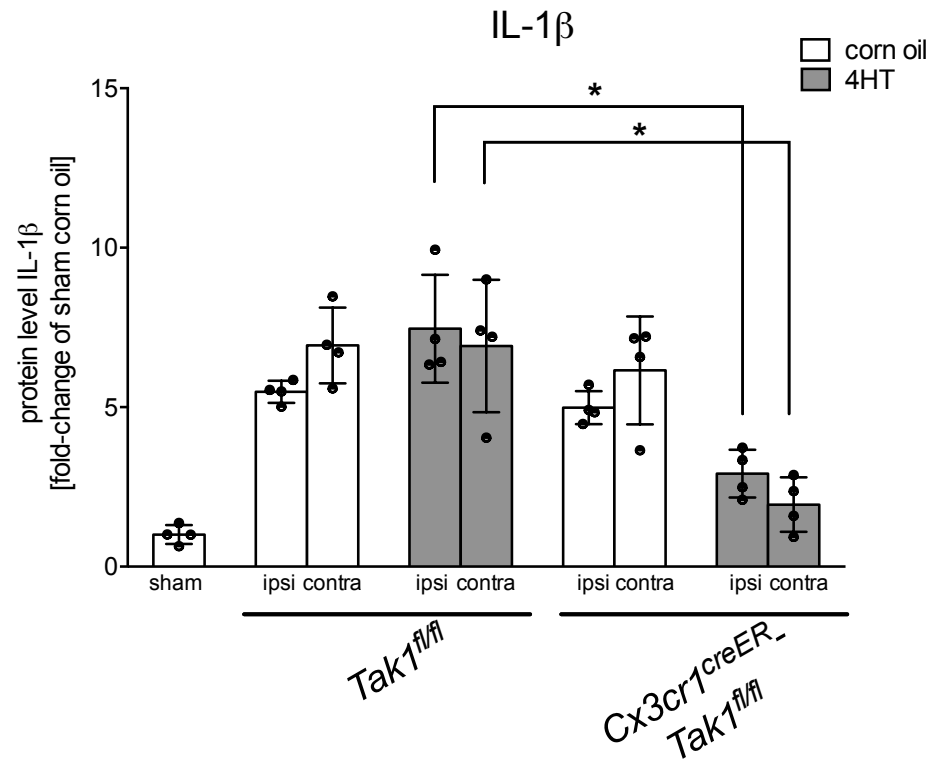

B

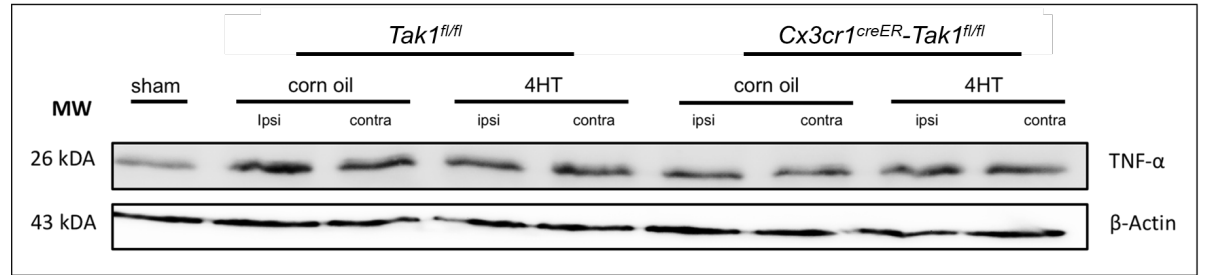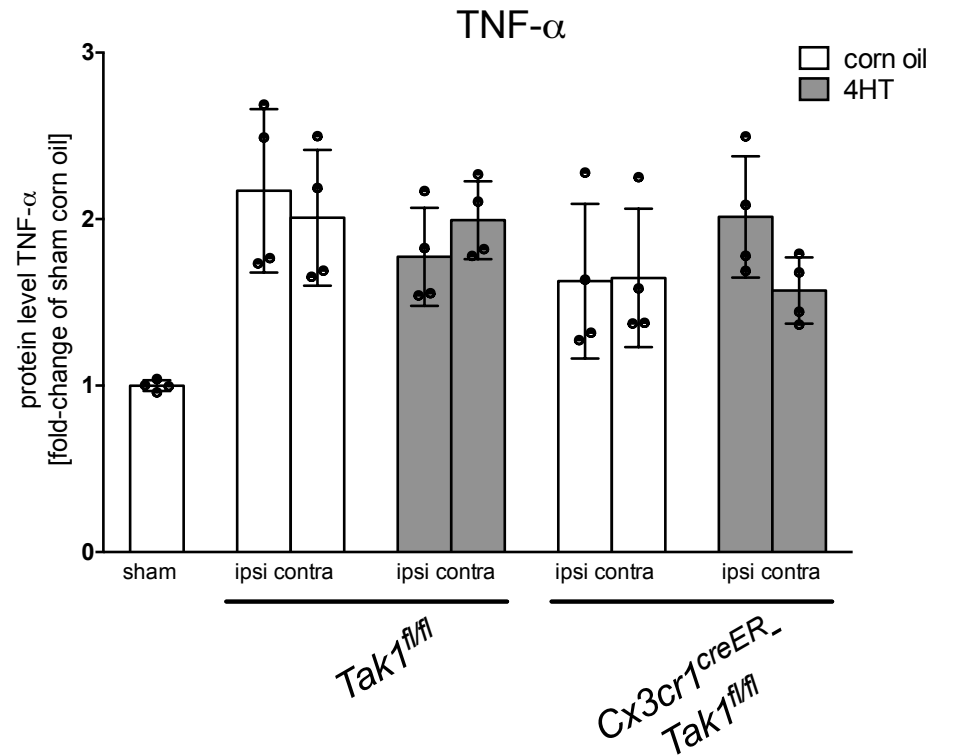

Supplement: Supplementary file 5 — Western Blot analysis of pro-inflammatory IL-1β and TNF-α in peri-infarct zone 72 h after tMCAo. (A,B) Protein-levels of IL-1β and TNF-α in peri-infarct zone were detected by immunoblotting (n = 6). β-Actin served as loading control. Quantification of protein-levels by densidometric analysis. All data are presented as Mean ± SD, individual data points are shown. Intergroup differences were tested by ANOVA three-way. (PDF 353 kb) [file 109_2020_1916_MOESM5_ESM.pdf]

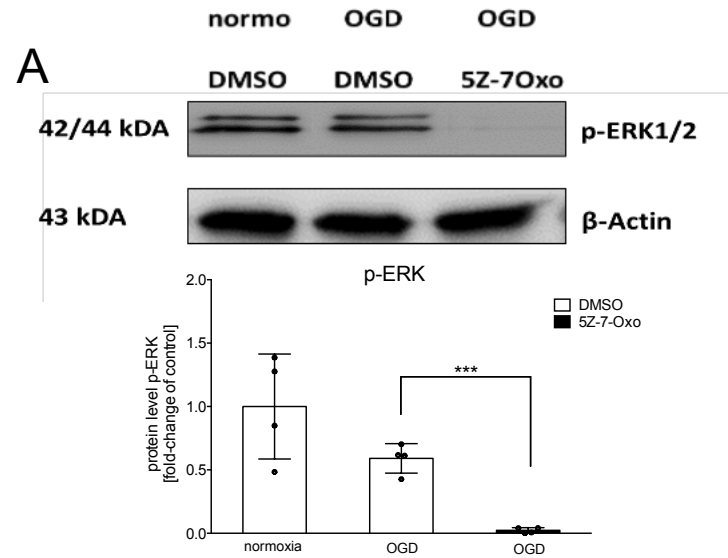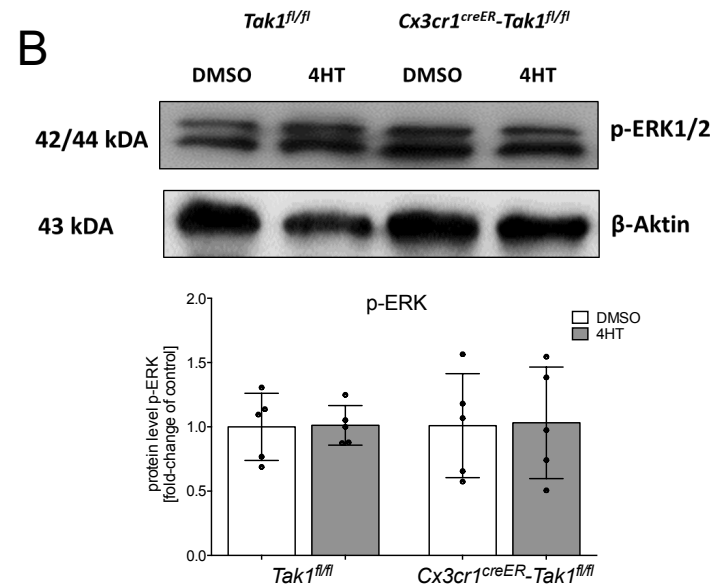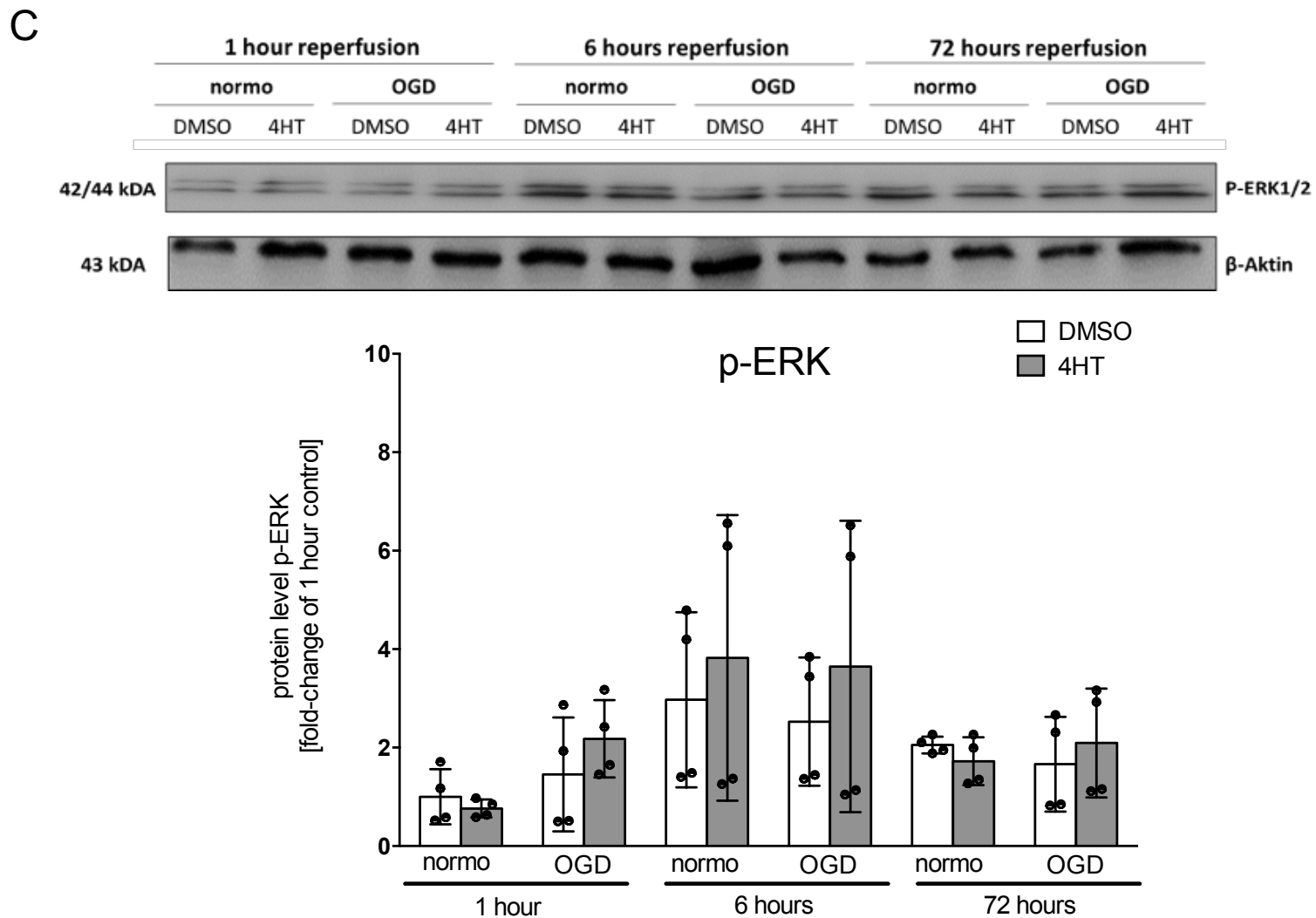

Supplement: Supplementary file 7 — Glial P-ERK levels do not change in response to OGD in mixed glial cell cultures. (A,B,C) Protein levels of activated ERK1/2 were detected by immunoblotting after 3 h of OGD and different timepoints after OGD (n = 4). β-Actin served as a loading control. Quantification of protein levels by densidometric analysis. (PDF 1471 kb) [file 109_2020_1916_MOESM7_ESM.pdf]

**A**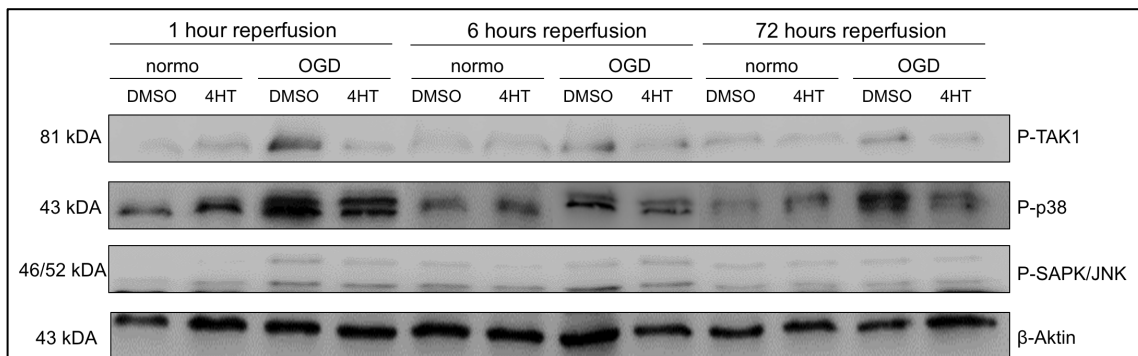**B**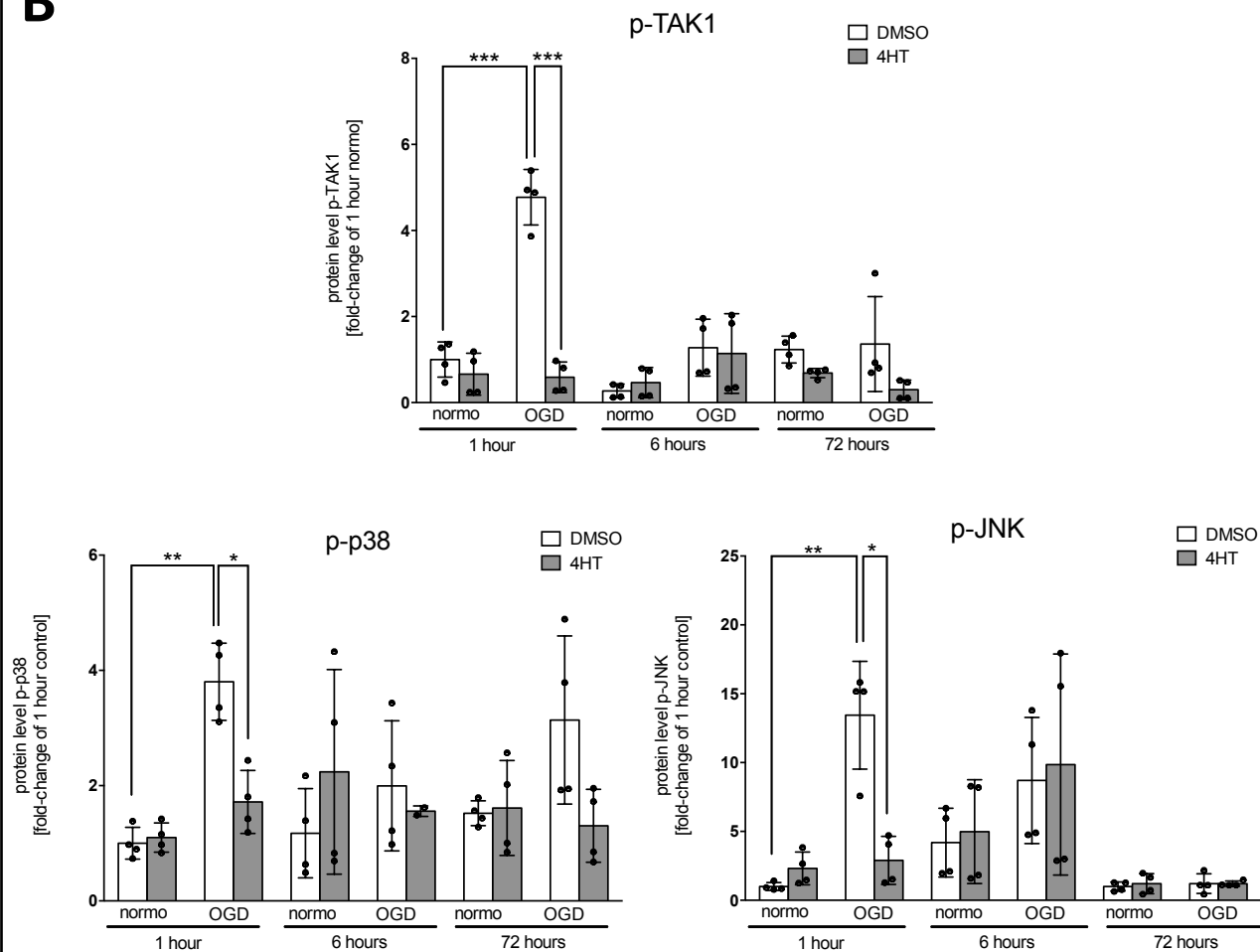

Supplement: Supplementary file 8 — TAK1 activation occurs in early phase of Oxygen-glucose-deprivation. (A) Protein levels of activated TAK1, p38, ERK1/2, JNK were detected by immunoblotting after 3 h of OGD and different timepoints after OGD (n = 4). β-Actin served as a loading control. (B) Quantification of protein levels by densidometric analysis. All data are presented as Mean ± SD, individual data points are shown. Intergroup differences were tested by ANOVA three-way. (PDF 603 kb) [file 109_2020_1916_MOESM8_ESM.pdf]

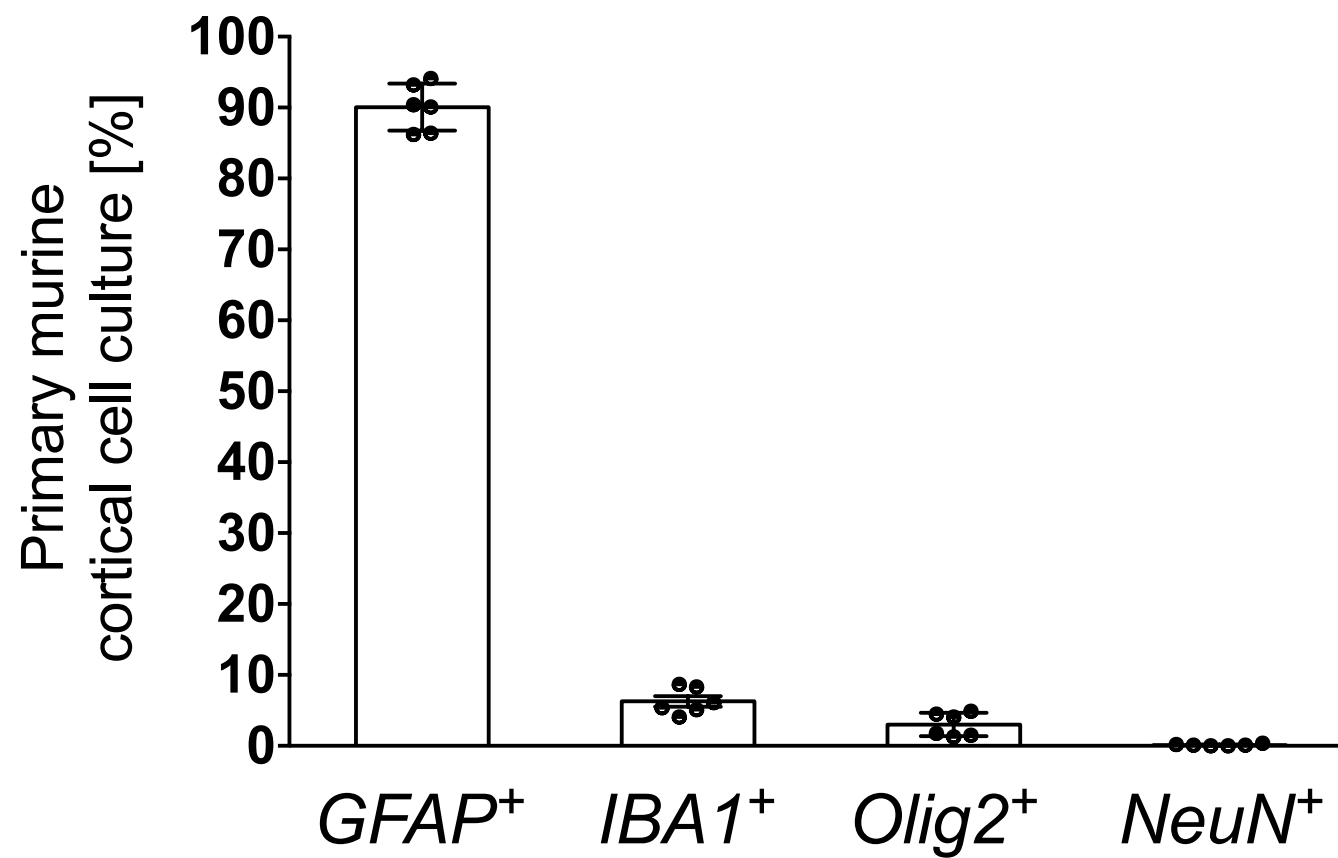

Supplement: Supplementary file 9 — Immunocytochemical staining of primary murine cortical cell culture of six independent preparations revealed 90.1% (± 3.3%) astrocytes, 6.3% (± 1.84%) microglia and 3% (± 1.7%) oligodendrocytes were found. We did not detect NeuN positive cells in our P2 cells. (PDF 36 kb) [file 109_2020_1916_MOESM9_ESM.pdf]
