# Supplementary material for: Microglial-specific depletion of TAK1 is neuroprotective in the acute phase after ischemic stroke
Source: J Mol Med (Berl). 2020 May 7;98(6):833–47. doi: 10.1007/s00109-020-01916-9 (PMC7297861; doi:10.1007/s00109-020-01916-9)
Supplement: Supplementary file 6 — TAK1-Inhibition reduced cell death rates in the first hour after OGD. (A) Schematic illustration of primary glial culture preparation, cell treatment and OGD-procedure. (B) LDH-release in Tak1fl/fl and Cx3cr1creER-Tak1fl/fl glial cell cultures after 3 h of OGD followed by 1 h, 6 h and 72 h of reperfusion is shown. Cells were treated with vehicle medium DMSO (“-”), Tamoxifen (4HT “+”) or with 5-7-Oxozeaenol (=5-7-Oxo “+”) (n = 4). All data are presented as Mean ± SD, individual data points are shown. Intergroup differences were tested by ANOVA two-way. (PDF 144 kb) [file 109_2020_1916_MOESM6_ESM.pdf]

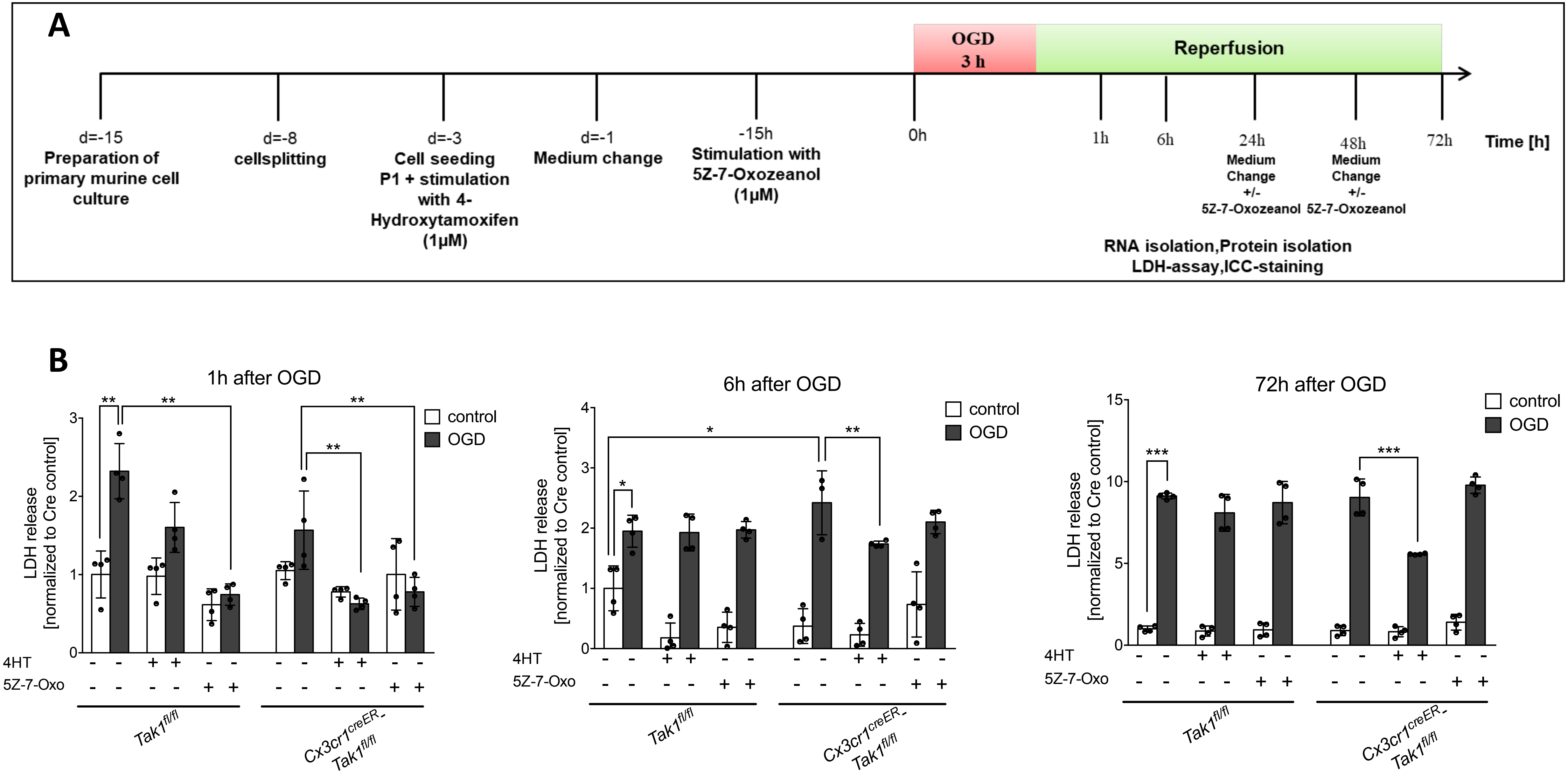
A
OGD 3 h
Reperfusion
d=-15 Preparation of primary murine cell culture
d=-8 cellsplitting
d=-3 Cell seeding P1 + stimulation with 4-Hydroxytamoxifen (1µM)
d=-1 Medium change
-15h Stimulation with 5Z-7-Oxozeanol (1µM)
0h
1h
6h
24h Medium Change +/- 5Z-7-Oxozeanol
48h Medium Change +/- 5Z-7-Oxozeanol
72h
Time [h]
RNA isolation,Protein isolation
LDH-assay,ICC-staining
B
1h after OGD
6h after OGD
72h after OGD
LDH release [normalized to Cre control]
control
OGD
4HT
5Z-7-Oxo
Tak1fl/fl
Cx3cr1creER-Tak1fl/fl
